# Stress-Induced Cell-Cycle Activation in Tip60 Haploinsufficient Adult Cardiomyocytes

**DOI:** 10.1371/journal.pone.0031569

**Published:** 2012-02-14

**Authors:** Joseph B. Fisher, Min-Su Kim, Steven Blinka, Zhi-Dong Ge, Tina Wan, Christine Duris, Desirae Christian, Kirk Twaroski, Paula North, John Auchampach, John Lough

**Affiliations:** 1 Department of Cell Biology Neurobiology and Anatomy and the Cardiovascular Center, Medical College of Wisconsin, Milwaukee, Wisconsin, United States of America; 2 Department of Pharmacology and Toxicology and the Cardiovascular Center, Medical College of Wisconsin, Milwaukee, Wisconsin, United States of America; 3 Division of Pediatric Pathology, Department of Pathology, Medical College of Wisconsin, Milwaukee, Wisconsin, United States of America; University of Massachusetts Medical, United States of America

## Abstract

**Background:**

Tat-interactive protein 60 (Tip60) is a member of the MYST family of histone acetyltransferases. Studies using cultured cells have shown that Tip60 has various functions including DNA repair, apoptosis and cell-cycle regulation. We globally ablated the *Tip60* gene (*Htatip*), observing that Tip60-null embryos die at the blastocyst stage (Hu et al. *Dev.Dyn*.238:2912;2009). Although adult heterozygous (Tip60^+/−^) mice reproduce normally without a haploinsufficient phenotype, stress caused by Myc over-expression induced B-cell lymphoma in Tip60^+/−^ adults, suggesting that Tip60 is a tumor suppressor (Gorrini et al. *Nature* 448:1063;2007). These findings prompted assessment of whether Tip60, alternative splicing of which generates two predominant isoforms termed Tip60α and Tip60β, functions to suppress the cell-cycle in adult cardiomyocytes.

**Methodology/Principal Findings:**

Western blotting revealed that Tip60α is the predominant Tip60 isoprotein in the embryonic heart, transitioning at neonatal stages to Tip60β, which is the only isoprotein in the adult heart wherein it is highly enriched. Over-expression of Tip60β, but not Tip60α, inhibited cell proliferation in NIH3T3 cells; and, Tip60-haploinsufficient cultured neonatal cardiomyocytes exhibited increased cell-cycle activity. To address whether Tip60β suppresses the cardiomyocyte cell-cycle in the adult heart, hypertrophic stress was induced in Tip60^+/+^ and Tip^+/−^ littermates via two methods, Myc over-expression and aortic banding. Based on immunostaining cell-cycle markers and western blotting cyclin D, stress increased cardiomyocyte cell-cycle mobilization in Tip60^+/−^ hearts, in comparison with Tip60^+/+^ littermates. Aortic-banded Tip60^+/−^ hearts also exhibited significantly decreased apoptosis.

**Conclusions/Significance:**

These findings provide evidence that Tip60 may function in a tumor suppressor pathway(s) to maintain adult cardiomyocytes in replicative senescence.

## Introduction

Tip60 is a member of the MYST family [1]. MYST is an acronym for the names of the founding family members MOZ, YBF2, SAS2, and Tip60. MYSTs are related by possession of a histone acetyl transferase (HAT) domain, which acetylates both histone [2], [3] and non-histone proteins in a variety of cellular processes [4]. Tip60 is intriguing because it also contains a chromodomain, which is similar to gene repressive domains in proteins such as heterochromatin protein-1. Most functions attributed to Tip60 involve co-activation or co-repression of gene promoters in context-dependent fashion [1]; for example we previously reported that Tip60 and serum response factor (SRF) co-activate the ANF promoter [5]. However, Tip60 is evidently pleiotropic, based on genome-wide siRNA screens indicating that it is one of six “hub” proteins that regulate multiple signal transduction pathways [6], as well as among six proteins that induce p53-dependent cell-cycle inhibition [7]. That Tip60 is a vital molecule is consistent with our finding that global ablation of the gene encoding Tip60 (*Htatip*) results in 100% penetrant embryolethality at the blastocyst stage [8].

Evidence indicates that Tip60 may inhibit the cell-cycle. In addition to its presumed function of inducing p53-dependent cell-cycle regulation [7], Tip60 and p53 have been shown to co-activate the gene encoding p21 [7], [9], [10], [11], [12], which is a well-documented inhibitor of Cdk-induced cell-cycle transit. Tip60 also inhibits mesangial cell proliferation [13]. Most relevant to this paper, Tip60 has been shown to function as a tumor suppressor in B-lymphocytes [14]. In this regard, Tip60 also promotes apoptosis, a function noted a decade ago [15] and recently documented [10], [11], [13], [16], [17]; interestingly, Tip60 may induce apoptosis by acetylating p53, which activates the latter's pro-apoptotic function [10], [16].

In the developing mouse heart, cardiomyocyte DNA synthesis, karyokinesis and cytokinesis become arrested by the third postnatal week [18]. However, contrary to previous dogma that adult cardiomyocytes cannot proliferate, recent evidence indicates that adult cardiomyocytes may re-enter the cell-cycle and divide [19], [20], [21]. Moreover, although adult cardiomyocyte renewal appears to occur at a very low rate [22], this notion was recently challenged [23]. It has been speculated that unknown inhibitory factors, operating in multiple pathways, combinatorially function to maintain adult cardiomyocytes in a state of post-replicative senescence [24]. We previously reported that Tip60 is expressed in the developing heart [5], [25]; however, because subsequent work revealed that Tip60-null mice die at the blastocyst stage and that Tip60-heterozygous mice do not have a haploinsufficient phenotype at adult stages [8], Tip60's role in embryonic and adult cardiomyocytes could not be assessed. However, In adult Tip60-heterozygous B-lymphocytes, imposition of Myc-induced stress was recently shown to induce lymphomagenesis, revealing a haploinsufficient phenotype accompanied by diminished cell-cycle control [14]. This prompted us to consider whether stress of cardiac hypertrophy, induced by Myc over-expression or aortic banding, could induce a Tip60 haploinsufficient phenotype in adult cardiomyocytes. We report that hypertrophy induced in Tip60^+/−^ adult hearts by either stressor causes increased cardiomyocyte cell-cycle activity, which is accompanied by reduced apoptosis in aortic-banded hearts. These findings support the notion that Tip60 functions to maintain cell-cycle inhibition, and promote apoptosis, in the adult myocardium.

## Materials and Methods

All experimental procedures are described in detail in File S1.

### Animals and Genotyping

All aspects of this investigation adhered to the National Institutes of Health (NIH) Guide for the Care and Use of Laboratory Animals (NIH Pub. No. 85-23, Revised 1996). All protocols, which are described in the corresponding author's Animal Use Application (AUA) #225, were approved by the Medical College of Wisconsin Institutional Animal Care and Use Committee (IACUC). The Medical College of Wisconsin has an Animal Welfare Assurance on file with the Office of Laboratory Animal Welfare (A3102-01). Targeting of the *Htatip* gene, preparation of the mouse Tip60 knockout line, and PCR/Southern blot genotyping were recently described in detail [8]. The transgenic mouse line *αMHC-MycER* was generously provided by Dr. Robb MacLellan (UCLA School of Medicine [26];); detection of the MycER transgene was performed using the following primer pair: 5′- TTGCGGAAACGACGAGAACAGT -3′ [fwd] and 5′- CTGCTAGGTTGGTCAATAAGCC -3′ [rev].

### Induction of Cardiac Hypertrophy by Myc Over-expression

Mice containing the *αMHC-MycER* transgene were bred into the wild type (Tip60^+/+^) and heterozygous (Tip60^+/−^) backgrounds. All experiments were performed using 2–3 month-old adult Tip60^+/+^ and Tip60^+/−^ littermates. The MycER fusion protein, which is specifically and constitutively expressed in cardiomyocytes, remains in the cytoplasm until mice are treated with 4-hydroxytamoxifen (4-OHT; Sigma #H7904; 1.0 mg/mouse/day i.p. for 7 days), whereupon MycER translocates to the nucleus to induce expression of Myc-dependent genes, resulting in hypertrophy [27]. In one set of experiments mice were also treated with BrdUrd (Sigma #B9285, 1.25 mg/mouse i.p.) during the last two days (days 6–7) of 4-OHT treatment. On the day after completing 4-OHT/BrdUrd treatment, mice were euthanized and hearts were removed for RNA, protein and histological analyses.

### Induction of Cardiac Hypertrophy by Aortic Banding

The transverse aorta of 12–14 week-old adult mice was ligated with 7-0 prolene to achieve a luminal diameter of ∼0.4 mm. After two weeks, this resulted in a 45% increase in left ventricular mass.

### Culture of Cardiomyocytes and Transfection of NIH3T3 Cells

Neonatal cardiomyocytes were enzymatically isolated using the Worthington Neonatal Cardiomyocyte Isolation System, followed by a Percoll purification step and pre-plating to remove fibroblasts. Cultured NIH3T3 cells were transfected (using Lipofectamine 2000) with p3X-CMV-FLAG plasmid vectors encoding Tip60α and Tip60β cDNAs, as described in File S1.

### Semi-Quantitative RT-PCR

Performed using a primer pair that anneals to domains in exons 7 and 10 of the Tip60 gene, amplifying a 420 bp PCR product from both Tip60α and Tip60β cDNA.

### Western Blotting

Tip60α and Tip60β isoproteins were identified using a custom affinity-pure rabbit polyclonal antibody raised against peptide EGCRLPVLRRNQDNE, which is present in the N-terminus of all Tip60 isoproteins and is absent from all other proteins including the highly related MYST family member MOF. This antibody was characterized as described in File S1 and Figure S1. The Cyclin D2 antibody was purchased from Santa Cruz Biotech (#sc-593).

### Immunohistochemical and TUNEL

Labeling were performed using commercially available antibodies and reagents. Cardiomyocyte identity was determined by immunostaining sarcomeric α-actin (cytoplasm) and Nkx2.5 (nucleus). TUNEL was performed using the DeadEnd TUNEL kit (Promega); cells were scored as TUNEL-positive only if at least 50% of the nucleus was filled with signal. Within each heart, 1,500–5,000 cells were enumerated to calculate percentages of labeled cells, relative to total nuclei.

### Statistical significance

Determined using Student's t-Test (two-tailed, unpaired).

## Results

### Tip60 Isoprotein Transition during Heart Development and Maturation

**Figure 1:**
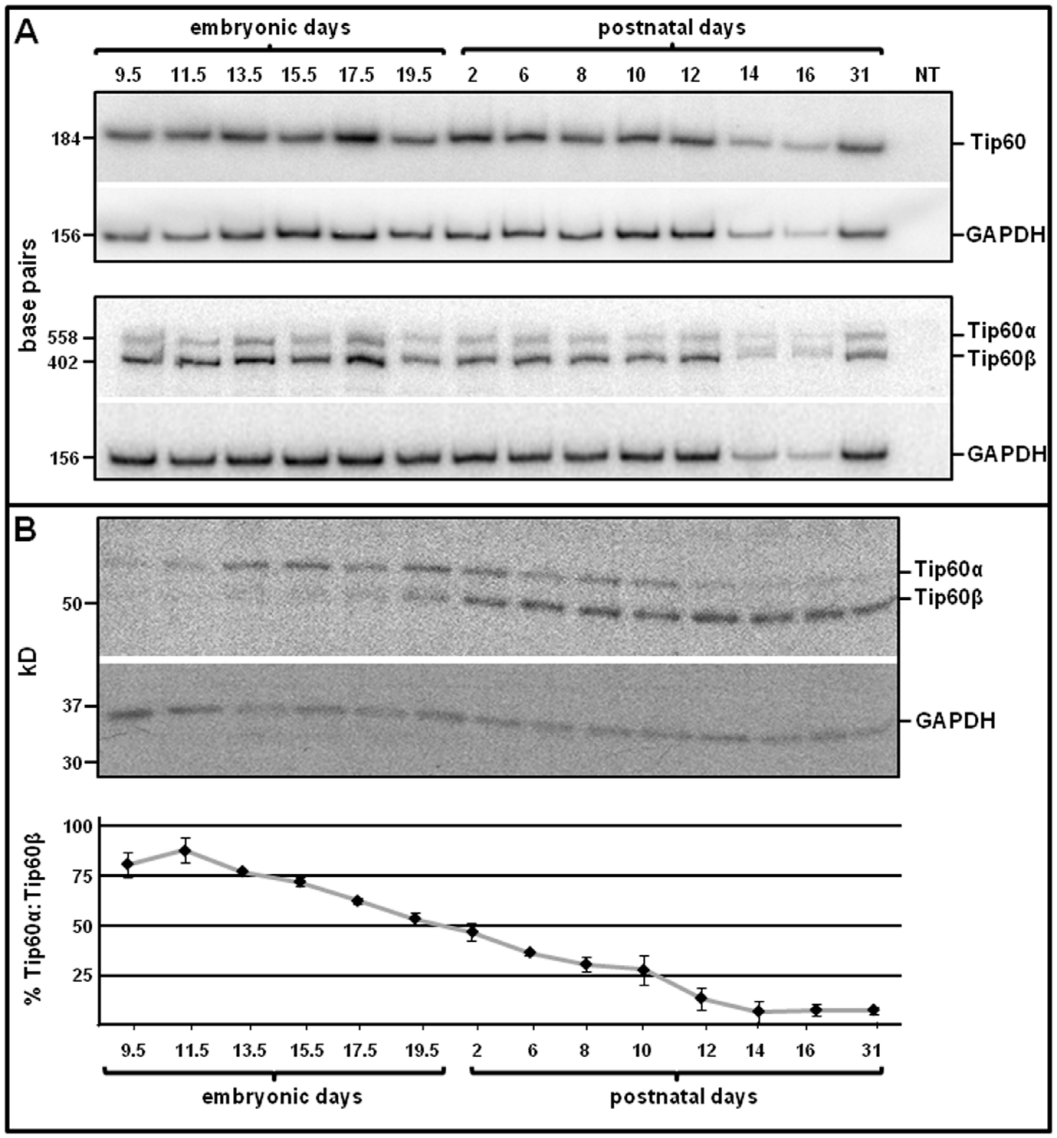
Tip60 Expression during Heart Development & Maturation. Total RNA and protein was purified at the indicated stages of development and subjected to semi-quantitative (**A**) RT/PCR and (**B**) western blotting analysis. **A**, the 184 bp band in the upper panel is amplified from all known isoforms of Tip60, whereas the 402 and 558 bp bands in the lower panel are respectively amplified from the Tip60 β and α isoforms. **B**, the upper panel is a western blot sequentially probed with antibodies recognizing Tip60 (α & β isoproteins were detected with the same antibody) and GAPDH; protein from individual hearts was evaluated at each postnatal day, whereas pools of three hearts each were evaluated at each embryonic day. The lower panel shows densitometric analysis in which each point indicates the mean of three (N = 3) independent western blot determinations. Error bars indicate ±SEM.

**Figure 2:**
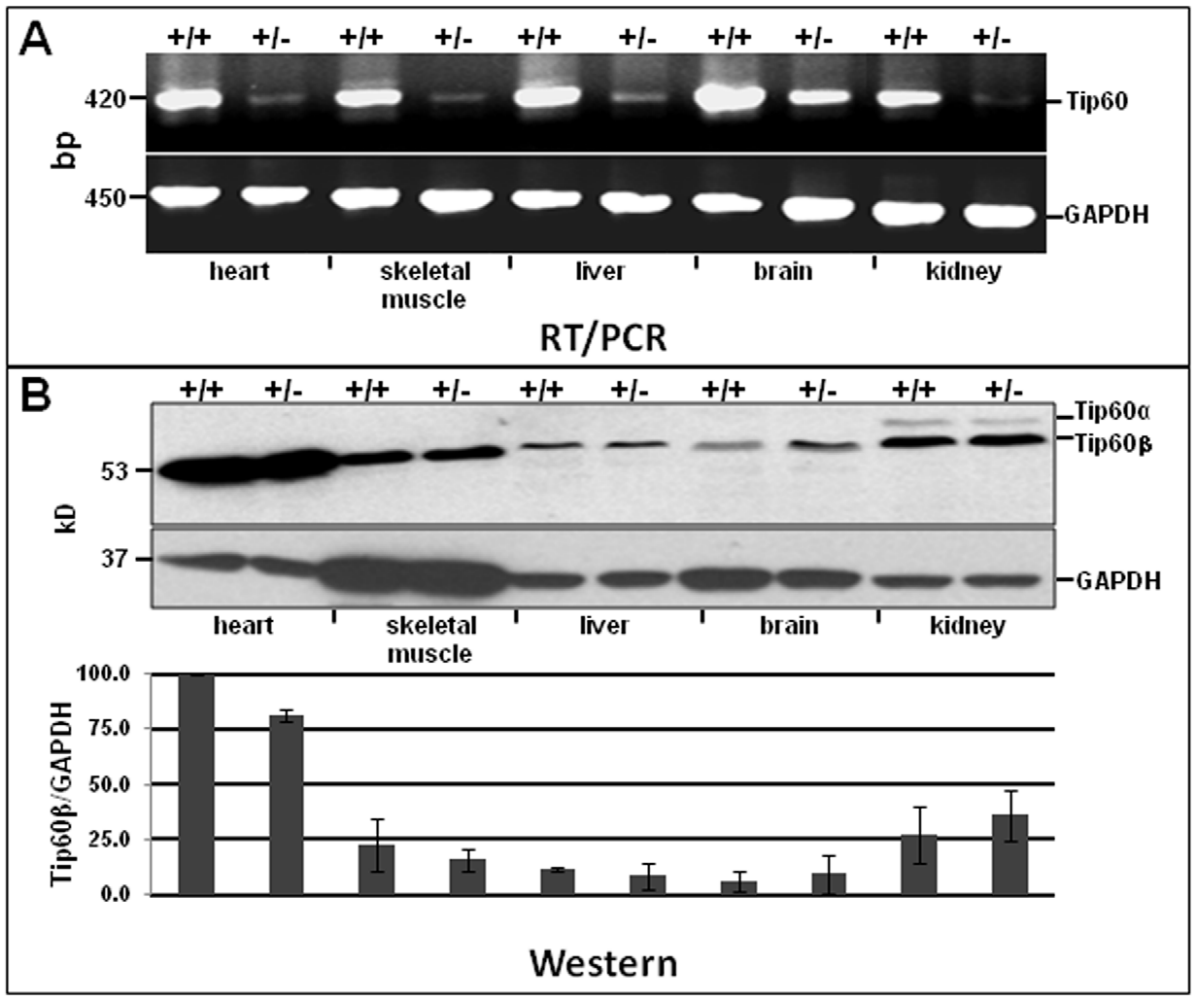
Tip60 Expression in Adult Organs. Tissues from 14 week-old adult Tip60^+/+^ and Tip60^+/−^ mice were processed for (**A**) semi-quantitative RT/PCR to assess transcript levels (of bulk Tip60 isoforms) in WT and Het tissues and (**B**) Western blotting to determine Tip60 protein levels. The primer pair used for RT/PCR (**A**) detected transcripts encoding both Tip60α and Tip60β; GAPDH was assessed as loading control. The bar graph in the lower part of **B** shows densitometric analysis of protein bands assessed from duplicate animals, normalized to GAPDH and expressed as a percentage of the most abundant band (in WT heart); vertical bars  =  range. +/+  =  WT; +/−  =  Het.

Tip60 exists as two major isoproteins, Tip60α (60 kD) and Tip60β (53 kD; also known as PLIP, [13]), which are alternate-splice products of the *Htatip* gene [28]. Using an antibody against the N-terminus of Tip60 (characterization described in File S1 and Fig. S1), western blotting was performed to assess relative isoprotein levels, from the earliest stages of heart development until the adult stage. As shown in Figure 1B, relative levels of Tip60α and Tip60β isoprotein markedly transitioned, from Tip60α enrichment at embryonic stages (Fig. 1B, E9.5–E19.5) to Tip60β enrichment at postnatal stages. By the 31^st^ postnatal day, Tip60β was the predominant Tip60 isoprotein in the heart (Fig. 1A), as well as in other adult tissues in which Tip60α was present at barely detectable levels (Fig. 2).

Figure 2 shows steady-state levels of Tip60 mRNA and protein in various wild-type and Tip60-heterozygous tissues of adult mice. Despite the presence of haploinsufficient levels of Tip60 mRNA (Fig. 2A), amounts of Tip60 protein in heterozygous tissues were similar to wild-type levels (Fig. 2B). This phenomenon, which has also been observed in B lymphocytes [14] and in cerebellum [29] of Tip60-heterozygous mice, suggests that absence of a haploinsufficient Tip60 phenotype is mediated by the ability of Tip60-heterozygous organs to maintain Tip60 protein at near-normal levels. It is noteworthy that among these tissues the adult heart exhibited the highest level of Tip60β protein.

To assess whether the isoprotein transition in Figure 1 reflected (i) a switch occurring in cardiomyocytes *per se* or (ii) increasing numbers of Tip60β-expressing non-myocytes in the developing heart, cardiomyocyte percentages in the embryonic and adult heart were determined by immunostaining Nkx2.5, a marker expressed in cardiomyocytes but not in other myocardial cells (fibroblasts, smooth muscle cells, endothelial cells). As shown in panels A–C of Figure S2, the percentage of cardiomyocytes in ventricular myocardium declined from ∼70% at embryonic day (ED) 14, to approximately 35% in the 10 week-old adult heart. It was therefore important to discern whether the predominance of Tip60β in the adult heart shown in Figures 1 & 2 reflected increased numbers of non-myocytes, or a cardiomyocyte-specific phenomenon. Hence, cells in two day-old neonatal and ten week-old adult hearts were isolated and separated into cardiomyocyte (CM) and non-myocyte (non-CM) fractions, followed by western blotting to detect Tip60α and Tip60β. As shown in panel D of Figure S2, the predominant isoproteins in neonatal cardiomyocytes and non-myocytes (most of which are presumably fibroblasts [30];) were, respectively, Tip60α and Tip60β. By contrast, in the adult heart, Tip60β was the major isoprotein in both cardiomyocytes and non-myocytes. Therefore, although a significant proportion of increasing Tip60β content during heart development was contributed by non-myocytes, Tip60α and Tip60β are, respectively, the major isoproteins in neonatal and adult cardiomyocytes.

### Tip60 May Inhibit Cell Proliferation

**Figure 3:**
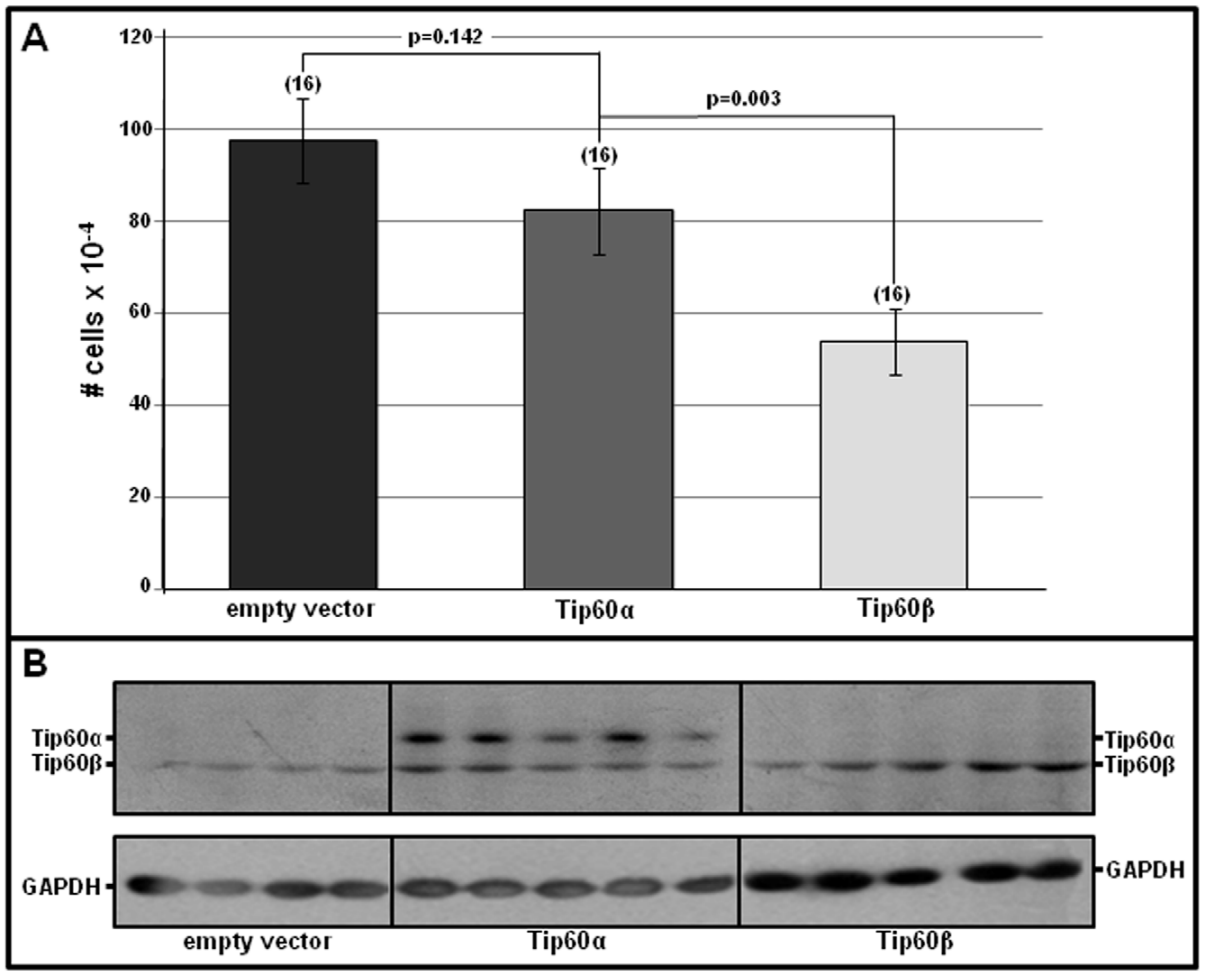
Over-Expression of Tip60β, but not Tip60α, Inhibits Cell Proliferation in NIH/3T3 Cells. Cultured NIH/3T3 cells were transfected with plasmid p3xFLAG-CMV-7.1 (empty vector), or with the same vector containing cDNA encoding either Tip60α or Tip60β. **A** shows the average cell number in each well of a 12-well plate, five days after transfecting the cells with plasmid. Error bars  =  ±SEM; p-values were calculated using Student's t-test (two-tailed, unpaired). **B** shows western blots confirming exogenous expression of Tip60α and Tip60β isoproteins.

**Figure 4:**
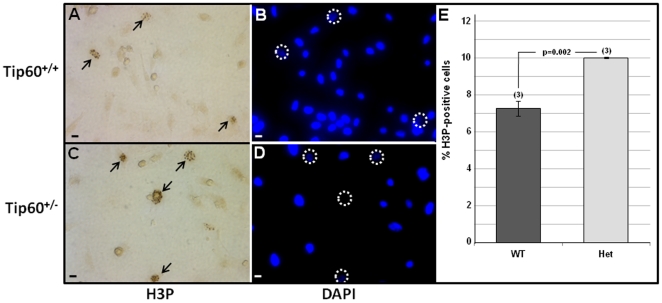
Increased Cell-Cycle Activity in Tip60^+/−^ Neonatal Cardiomyocytes. Cardiomyocytes were isolated from 2 day-old neonatal hearts and cultured on gelatin-coated dishes. When the cells were ∼60% confluent (72 hrs later) they were double-immunostained for phosphorylated histone-H3 (H3P) to detect M-phase cells (arrows in **A** & **C**) and for sarcomeric α-actin (not shown) to verify cardiomyocyte identity. Nuclei were stained with DAPI (**B**,**D**); H3P-labeled nuclei are encircled because DAPI is obscured DAB-stained nuclei. E shows percentages of H3P-positive neonatal cardiomyocytes, based on enumerating 1,000-2,000 cells in each dish; error bars  =  +/−SEM. Scale bars in **A**–**D** = 10 µm.

As noted above, previous findings have indicated that Tip60 may inhibit the cell-cycle in various cultured cells [7], [9], [10], [11], [12], [13] as well as *in vivo* B lymphocytes [14]. These findings, plus the Tip60 α to β isoprotein transition (Fig. 1) that occurs as cardiomyocytes withdraw from the cell-cycle [18], resulting in high Tip60β levels in adult cardiomyocytes (Fig. 2B & File S2E–F), suggested that Tip60β may inhibit the cardiomyocyte cell-cycle. Although the recently described Tip60 global knockout [8] was designed to assess Tip60 function in the heart, lethality of Tip60-null blastocysts, plus the absence of a robust haploinsufficient phenotype at later stages, prevented our ability to address this question directly. As alternative, two *in vitro* approaches were employed. First, the effect of over-expressing Tip60α and Tip60β encoded in transfected plasmids was evaluated in proliferating cells. This determination was performed using cultured NIH/3T3 cells rather than neonatal mouse cardiomyocytes, since the latter are very poorly transfected. As shown in Figure 3, despite the expression of approximately equal levels of plasmid-encoded Tip60α and Tip60β isoprotein (Fig. 3B), Tip60β, but not Tip60α, inhibited proliferation (Fig. 3A). As an aside it was also noted that cells transfected with Tip60α exhibited increased levels of endogenous Tip60β, which may have induced the slight, non-significant reduction of proliferation observed in Figure 3A (see Discussion). The second approach exploited the recently demonstrated ability of cardiomyocytes to respond to stress of explantation into the cell culture environment by undergoing dedifferentiation and proliferation [31]. Hence, we asked whether the extent of proliferation in cultured Tip60-heterozygous neonatal cardiomyocytes was increased, relative to that in Tip60-wild type cardiomyocytes. In this determination, neonatal myocytes from two day-old Tip60^+/+^ and Tip60^+/−^ hearts were purified and cultured, followed three days later by immunostaining phosphorylated histone H3 (H3P) and sarcomeric α-actin. As shown in Figure 4, in comparison with Tip60^+/+^ cardiomyocytes, the percentage of M-phase (H3P-positive) cells in Tip60^+/−^ cardiomyocytes was >25% increased (Fig. 4E), indicating a Tip60 haploinsufficient phenotype of increased cardiomyocyte proliferation.

### Myc Induces Cell-Cycle Transit in Tip60-Haploinsufficient Adult Cardiomyocytes

Taken together, the findings in Figures 1,2,3,4 suggest that Tip60β may inhibit the cardiomyocyte cell-cycle. This prompted assessment of the extent to which stress of cardiac hypertrophy induced by Myc alters the cell-cycle in wild type and Tip60-heterozygous adult cardiomyocytes. For these experiments, transgenic *αMHC-MycER* mice were bred into Tip60^+/+^ and Tip60^+/−^ backgrounds. Upon administration of tamoxifen (4-OHT), the MycER fusion protein, which is restricted to cardiomyocyte cytoplasm, translocates to the nucleus where it activates genes promoting cell-cycle induction [26]. As anticipated, steady-state levels of MycER mRNA were similar in Tip60^+/+^ and Tip60^+/−^ adult hearts (Fig. S3 A,B), and, administration of 4-OHT induced similar levels of cardiac hypertrophy in these genotypes (Fig. S3C).

**Figure 5:**
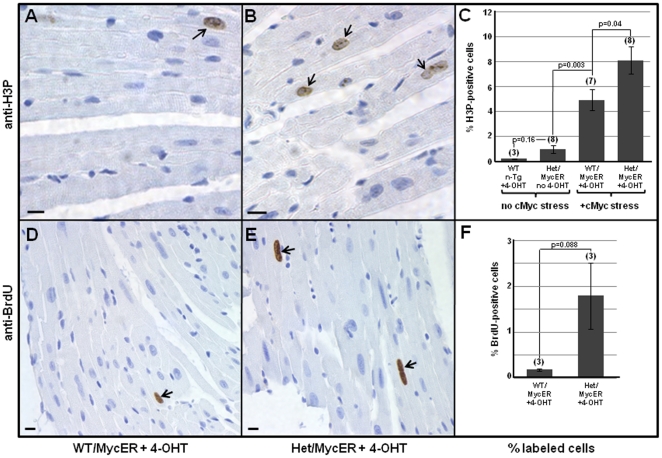
Tip60-Haploinsufficiency Augments 4-OHT-Induced Induction of Cell-Cycle Activity in MycER Transgenic Hearts. Eight week old MycER transgenic Tip60 wild-type (WT) and heterozygous (Het) mice were induced with 4-OHT for seven days and assessed for cell-cycle activity by immunostaining phosphorylated histone H3 (H3P; arrows in **A**,**B**). This was verified by immunostaining BrdU-incorporated nuclei (arrows in **D**,**E**). Percentages of labeled cells were determined by evaluating at least 5,000 (**C**) or 2,500 (**F**) hematoxylin-stained nuclei for H3P or BrdU antigen, respectively. (N)  =  number of hearts; vertical bars  =  ±SEM; p-values were calculated using Student's t-Test (two-tailed, unpaired). The scale bar in all images  =  10 µm. (Note: A control utilizing WT-MycER mice demonstrated that 4-OHT had no effect on these parameters [26]).

As shown in Figure 5 (A–C), phosphorylated histone H3 (H3P) immunostaining revealed that whereas cell-cycle activity was slightly but not significantly increased in the absence of cMyc-induced stress, induction of cMyc with 4-OHT significantly increased cell-cycle activity in WT and especially in Het myocardial cells (Fig. 5C). This difference was verified by immunostaining BrdUrd, which was provided on the last two days of the seven day treatment period (Fig. 5 D–F). This haploinsufficient effect occurred despite western blot densitometry indicating that levels of Tip60β protein in Tip60^+/−^ hearts were decreased only ∼20% (Fig. S4 B,D). Although non-significant, this was consistent with echocardiographic trends indicating that LV posterior wall thickness and LV mass were increased in Tip60-heterozygous hearts treated with 4-OHT (Fig. S5). The increase in cycling cells in Tip60^+/−^ relative to Tip60^+/+^ hearts was accompanied by a slight but significant increase in density of myonuclei in the myocardium (determined by immunostaining Nkx2.5), suggestive of karyokinesis; however, there was no evidence of cytokinesis, based on absence of aurora kinase B staining (not shown).

**Figure 6:**
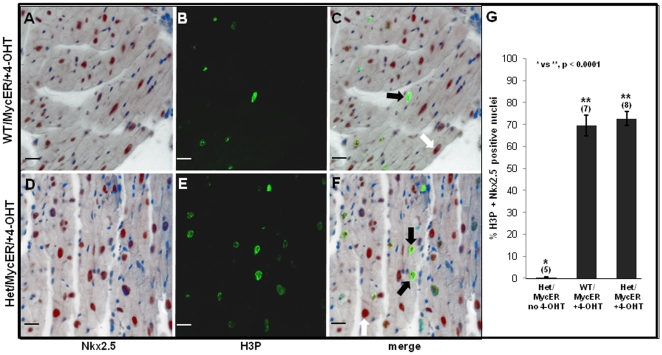
Most H3P-Positive Nuclei are in Cardiomyocytes. Sections from hearts processed in Figure 5 were double-immunostained for phosphorylated H3P and Nkx2.5 to determine cardiomyocyte identity. **A** & **D** show Nkx2.5 staining (nucleus-specific brown DAB reaction product) in cardiomyocyte nuclei, as distinct from smaller non-myocyte nuclei in which only (blue) hematoxylin counter-stain is seen. **B** & **E** show H3P fluorescent green signal in the same nuclei. **C** and **F** are merged images of A–B and D–E, respectively. In **C** & **F**, dark arrows denote nuclei double-stained for Nkx2.5 and H3P; white arrows denote nuclei expressing only Nkx2.5. **G** summarizes results from enumerating a minimum of 150 H3P-positive nuclei per heart for co-localization of Nkx2.5. Error bars  =  ±SEM; statistical significance was determined by Student's t-Test (two-tailed, unpaired). Scale bars  = 20 µM.

It was important to assess whether the H3P-positive nuclei in 4-OHT-treated hearts were in cardiomyocytes or in non-myocytes. To address this question, H3P immunostaining was performed in combination with Nkx2.5, which is expressed only in cardiomyocyte nuclei. This revealed that most (70–75%) of the H3P-positive nuclei in 4-OHT-treated hearts were in cardiomyocytes, regardless of phenotype (Fig. 6). Assessment of TUNEL-positive cells in adjacent sections did not detect an apoptotic effect in either genetic background (not shown).

**Figure 7:**
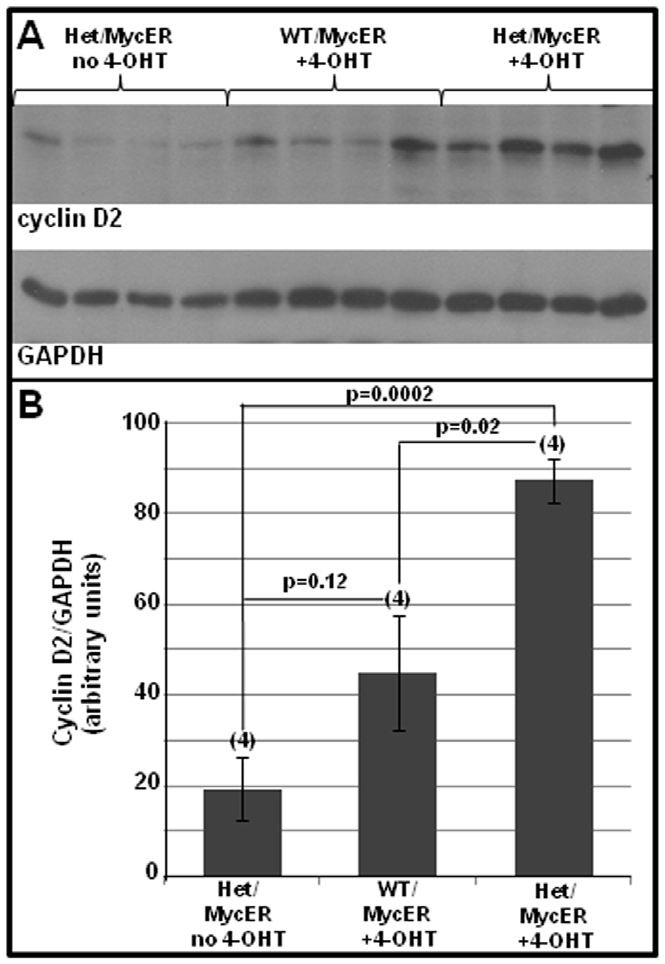
Tip60-Haploinsufficiency Increases 4-OHT-Induced Expression of Cyclin D2. Eight week old MycER transgenic Tip60 wild-type (WT) or heterozygous (Het) mice were induced with 4-OHT for seven days and assessed for cyclin D2 by western blotting. **A** is a western blot showing cyclin D2 and GAPDH levels in non-stressed (no 4-OHT) and stressed (+4-OHT) WT and Het myocardium. Each lane contained 10 µg total protein from separate hearts. **B** shows densitometry of the bands in **A**, with cyclin D2 normalized to GAPDH. Numbers (N) in parentheses indicate numbers of hearts evaluated. Vertical bars  =  ±SEM; p-values were calculated using Student's t-Test (two-tailed, unpaired).

To correlate the microscopic evidence for cell-cycle activation (Figs. 5,6) with an early biochemical marker, western blotting of cyclin D2 was performed, revealing significantly higher levels of cyclin D2 protein in Tip60^+/−^ hearts compared with Tip60^+/+^ hearts (Fig. 7).

### Aortic Banding Induces Cell-Cycle Transit in Tip60-Haploinsufficient Adult Cardiomyocytes

**Figure 8:**
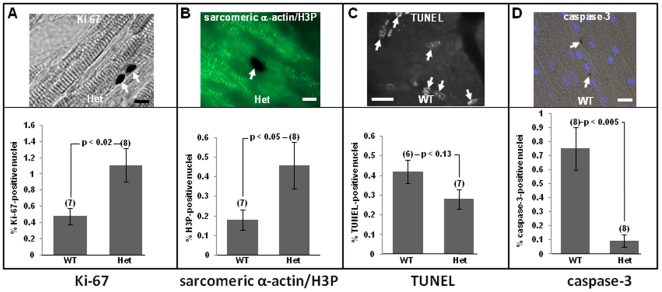
Induction of Cell-Cycle Activity and Reduced Apoptosis in Aortic-Banded Mice are Enhanced by Tip60-Haploinsufficiency. Mice were subjected to trans-aortic constriction (TAC) for two weeks. Panels **A-B** show Ki-67 (**A**) and phosphorylated histone H3 (H3P; **B**), which are significantly increased in Tip60-haploinsufficient myocardium. Panel **C** (TUNEL) shows a trend toward, and **D** (caspase-3 staining) demonstrates a significant reduction in, apoptosis in heterozygous myocardium. In **C**, cells were scored as TUNEL-positive if at least 50% of the nucleus contained signal. The microscopic images were selected as representative of the staining quality used to obtain data in the bar graphs. At least 1,500 DAPI-stained nuclei were enumerated in each heart for cell-cycle or apoptotic markers. (N)  =  number of hearts evaluated; vertical bars  =  ±SEM; statistical significance was via Student's t-Test (two-tailed, unpaired). Arrows in **A**–**C** denote labeled nuclei; arrows in **D** denote peri-nuclear loci of caspase-3. Bars in **A** = 25 µm; **B** = 15 µm.; **C**–**D** = 40 µm.

To confirm the findings in Figures 5,6,7, non-transgenic Tip60^+/+^ and Tip60^+/−^ mice were subjected to an alternative stressor, aortic banding, which induces cardiac hypertrophy within two weeks. As shown in Figure 8A, the percentage of Ki-67-immunostained cells, indicative of interphase cell-cycle transit, was increased over two-fold in banded Tip60^+/−^ hearts, in comparison with banded Tip60^+/+^ littermates. To assess whether cycling myocardial cells in banded hearts could progress into M-phase, adjacent sections were immunostained for H3P. This revealed that numbers of H3P-positive cells in Tip60^+/−^ myocardium were 2-3-fold increased in comparison with Tip60^+/+^ myocardium (Fig. 8B); moreover, co-immunostaining sarcomeric α-actin, as well as presence of cross-striated myofibrils, indicated that H3P-positive nuclei were within cardiomyocytes (Fig. 8B). Because physically-induced hypertrophy is known to increase the incidence of apoptosis in myocardial cells [32], and because Tip60 is a pro-apoptotic molecule [10], [15], [16], we assessed whether apoptosis was diminished in aortic-banded Tip60-heterozygous hearts. TUNEL-labeling revealed that apoptotic cells were ∼40% reduced in Tip60^+/−^ myocardium (Fig. 8C), while caspase-3 labeling (Fig. 8D) revealed an eight-fold decline. As in Myc-induced mice, these effects were observed under conditions in which Tip60β protein levels were only slightly reduced (−18%) as shown in Fig. S4 A,C).

## Discussion

Figure 1B demonstrates that the relative content of Tip60 isoproteins changes during heart development, from Tip60α enrichment during embryonic stages, to Tip60β enrichment at adult stages. Although semi-quantitative RT/PCR determinations (Fig. 1A) suggest that Tip60β mRNA is predominant at all stages, this may reflect relative primer pair inefficiency, and/or the possibility that the isoprotein transition is regulated at the level of translation. Because the western blot in Figure 1B is representative of three independent determinations, we believe that this transition represents a bonafide developmental event, significance of which is currently unknown. The only feature distinguishing Tip60α from Tip60β is that the 52 amino acid peptide encoded by exon 5 of the *Htatip* gene is absent from Tip60β, resultant from an alternative splice [28]. Although functional domains within exon 5 have not been characterized, one noteworthy feature is its inclusion of a hydrophobic domain (LPIPVQITLRFNL) suggestive of a nuclear export signal (NES). It would be interesting to assess whether absence of this motif in Tip60β prevents its ability to translocate from nucleus to cytoplasm, a previously documented behavior of Tip60 [33], [34]. Although this predicts that Tip60β would be confined to the nucleus of adult cardiomyocytes, a possibility with interesting functional implications, unavailability of a suitable antibody for Tip60 immunostaining has precluded immunolocalization.

Cardiac fibroblasts, which are the predominant non-myocyte cell type in the adult heart, begin to appear in the mouse heart after embryonic day 12 (ED 12). To estimate fibroblast contribution to the Tip60α→Tip60β isoprotein transition, cardiomyocyte percentages in fetal (ED14.5) and adult (10 wk old) mouse myocardium were assessed by immunostaining Nkx2.5, levels of which are intense in nuclei of embryonic/fetal cardiomyocytes and remain appreciable at adult stages. The >2-fold decline in 10 week-old adult hearts (Fig. S2C), which contain ∼35% cardiomyocytes, is consistent with percentages (30–35%) recently observed by subjecting isolated myocardial cells to flow cytometry [30]. Although this suggested that increasing numbers of non-myocytes, predominantly cardiac fibroblasts, may explain the transition to Tip60β predominance in the adult heart, western blotting of isolated myocyte and non-myocyte populations revealed that Tip60α expression, which was present in both cell types during the neonatal stage, was extinguished in both by the adult (8 week-old) stage (Fig. S2D). Hence, the transition of cardiomyocytes from a proliferating to a non-proliferative population during the neonatal stage is accompanied by a transition from Tip60α predominance to Tip60β near-exclusivity. Curiously, this transition occurs concomitant with the transition from regeneration-competent to regeneration-incompetent myocardium as recently described [35]. While the significance of this transition remains speculative at this point, the possibility that Tip60α, but not Tip60β, is permissive for cell proliferation is consistent with the findings in Figure 3; in this regard, whereas the unexpectedly increased levels of Tip60β in cells transfected with Tip60α slightly reduced proliferation, we suggest that more extensive reduction was prevented by the large amount of Tip60α present. Finally it is noteworthy that unlike fetal isoproteins that are re-expressed in adult hearts consequent to cardiac hypertrophy [26], [36], Tip60α was not re-expressed during hypertrophy induced by either aortic banding or Myc over-expression (Fig. S4).

Western blotting was used to compare Tip60β protein levels in selected adult tissues, including skeletal muscle, liver, brain, kidney and heart, the latter curiously exhibiting the highest levels of Tip60β protein (Fig. 2B). It is noted that Tip60β protein levels were only slightly reduced in Tip60-heterozygous heart tissue, despite marked depletion level of Tip60 mRNA (Fig. 2). This phenomenon has been observed by two other laboratories that utilized these mice [14], [29]; for example, over-expression of Myc in Tip60-heterozygous B-lymphocytes only slightly reduced Tip60 protein from wild-type levels [14]. Although hearts in both Myc-induced and aortic-banded Tip60-heterozygous mice exhibited reduced levels of Tip60β protein, the extent of reduction was not statistically significant (Fig. S4). This raises speculation that maintenance of Tip60 protein near normal levels in Tip60-heterozygous tissues is a cellular response designed to maintain vital processes that are mediated by this vital, pleiotropic protein [6], [8]. We considered that maintenance of Tip60 protein levels in Tip60-heterozygous tissues might be due to reduced levels of Mdm2 ubiquitin ligase, which regulates p53 and Tip60 levels via ubiquitin-mediated degradation [37]. However, western blotting indicated that Tip60-heterozygous heart tissue contained normal, wild-type levels of Mdm2 protein (not shown); similarly, levels of Mdm2 mRNA are similar in wild-type and Tip60-heterozygous B-lymphocytes [14]. Other possible mechanisms by which Tip60 protein levels are maintained in heterozygous tissues, for example via increased translational efficiency in the heterozygous condition, are being considered.

It was previously shown that cardiac hypertrophy induced by pressure overload, but not by Myc over-expression, is accompanied by cardiomyocyte apoptosis [38], [39]. Therefore, our finding that MycER induction caused neither increased TUNEL labeling nor activated caspase-3 staining (not shown), in contrast to the apoptotic effects induced by aortic-banding (Fig. 8 C, D), were anticipated. This distinction has been interpreted to reflect the ability of Myc to protect cardiomyocytes from apoptosis [39]. It is noteworthy that the results shown in Figure 8 C,D extend the previously demonstrated pro-apoptotic effect of Tip60 in cultured cells [10], [15], [16] to the *in vivo* situation.

We recently reported that whereas Tip60-null embryos undergo early embryolethal arrest, heterozygotes do not exhibit an overt haploinsufficient phenotype at any stage [8]. Because Myc induces a Tip60-haploinsufficient phenotype in B-lymphocytes, consistent with Tip60 function as a tumor suppressor [14], we assessed whether stress of cardiac hypertrophy induced by Myc or aortic banding altered cardiomyocyte cell-cycle control. Conditional over-expression of Myc in cardiomyocytes of the adult heart (Figs. 5,6,7), or stress induced by cardiac overload (Fig. 8), induced cell-cycle mobilization that was more extensive in Tip60-heterozygotes than in wild-type littermates. Surprisingly, numbers of H3P-positive cells (Fig. 5C) were increased relative to BrdUrd-labeled cells (Fig. 5F). We speculate that this may reflect accumulation of H3P-positive cells in M-phase throughout the seven day induction with 4-OHT, whereas BrdUrd-positive cells were labeled during only the last two days of this interval. Because nuclei co-immunostained with H3P and Nkx2.5 (Fig. 6) did not exhibit nuclear disintegration, it is suggested that cycling cells accumulated in late G_2_ or early M-phase, and/or that they underwent endomitosis [40]. It is also possible that some cells underwent karyokinesis, as suggested by slightly increased numbers of myonuclei observed in Tip60-heterozygous myocardium; however, there was no evidence of cytokinesis, as revealed by absence of aurora-B-kinase staining (not shown). It was also surprising that numbers of BrdUrd-labeled cells in wild-type hearts subjected to Myc-induced stress were essentially nil in comparison with the 9-fold increase seen in Tip60-heterozygous hearts (Fig. 5F). It is conjectured that this indicates that a finite cohort of cells is released from G_0_ arrest during early phases of the stress response (i.e. prior to BrdUrd labeling) under normal (wild type) conditions, whereas Tip60-haploinsufficiency permits continuing release of an expanded cohort in stressed cardiomyocytes, which is detected by BrdUrd labeling during days 6–7. All these possibilities are under further investigation.

Considered in the context of Tip60's previously reported tumor suppressor function [14], the findings reported here provide evidence that Tip60 may participate in maintenance of cardiomyocyte proliferative senescence, perhaps in concert with proteins including retinoblastoma (Rb) and p130 since ablation of these causes similar effects [41], [42]. Interestingly, some of Tip60's functions are closely related to the tumor suppressor p53; for example, Tip60 and p53 co-activate the gene encoding the cell-cycle inhibitor p21 [7], [9], and, Tip60 acetylates p53 to activate the latter's pro-apoptotic function [10], [16]. Because adult cardiomyocytes contain high levels of Tip60β (Fig. 2) and relatively low levels of p53 [43], it is speculated that Tip60 may function as a p53 surrogate. The findings reported here add to the list of factors in proto-oncogene (periostin [20], neuregulin [21], Myc [38], cyclin-D2 [44]) and tumor suppressor (p38 MAP kinase [19], Rb/p130 [41], [42]) pathways that can be manipulated to induce cell-cycle activity in adult cardiomyocytes *in vivo*, followed in some instances by cytokinesis after placement in an *in vitro* environment [19], [20], [21]. The molecular mechanism by which Tip60 may regulate the cardiomyocyte cell-cycle remains to be elucidated. In this regard, participation of Tip60 in DNA repair via its chromodomain, which binds histone H3 trimethylated on lysine-9 (H3K9me3) to enable HAT domain-mediated acetylation of ATM [45], is of interest because the chromodomain protein HP-1 was recently shown to bind H3K9me3 during heterochromatin formation as cardiomyocytes become senescent [46]. It will therefore be intriguing to consider that Tip60 promotes heterochromatin formation in adult cardiomyocytes, resulting in repression of cell-cycle genes such as Rb and p130 [46]. In this regard inactivation of Rb and the Ink4a product ARF was recently shown to induce cell-cycle activation in differentiated skeletal myocytes [47]. And, Tip60 has been implicated in the mechanism of cellular senescence [7], a process regulated by p38 MAPK [48]. However, although involvement of Tip60 in the p38 MAPK pathway suggests that its down-regulation could, similar to the effect of inhibiting p38 MAPK, result in cardiomyocyte cytokinesis [19], we failed to detect aurora kinase B immunostaining in stressed Tip60^+/−^ cardiomyocytes.

In summary, evidence has accumulated that Tip60 is a vital molecule, as evidenced by the embryolethal effect of its total ablation at the blastocyst stage [8], and by the apparent need for heterozygous cells to maintain Tip60 protein at normal wild-type levels (this paper and [14], [29]); the latter is consistent with our observations that both Tip60 siRNA and shRNA reduce Tip60 mRNA but not protein content in cultured NIH3T3 cells (not shown). Although the findings reported here demonstrate, as in previous instances [14], [29], that stress induces a haploinsufficient phenotype, this is accompanied by only marginally decreased content of Tip60 protein (Fig. S4), which likely reflects the modest response seen in some determinations (for example, TUNEL, Fig. 8C). The mechanism by which Tip60 mRNA-depleted cells maintain Tip60 normal protein levels will be interesting to investigate. Of more immediate interest is the need to knock down Tip60 protein, to levels that permit cell survival while causing an unequivocal cardiomyocyte phenotype, since this will likely be required to elucidate the molecular mechanism of Tip60 function in the myocardium. This goal will hopefully be fulfilled by conditionally and specifically ablating the Tip60 gene in adult cardiomyocytes, using a line of mice containing LoxP-flanked alleles that is in final stages of preparation.

## Supporting Information

File S1 **This file describes details of the** **Materials and Methods** **used for this study.** These include animal identity, care and handling; plasmid descriptions; Tip60 antibody characterization; western blotting; antibodies employed & immunohistochemical protocols; semi-quantitative RT-PCR protocols; protocols for induction of cardiac hypertrophy by aortic banding; methods for isolation & culture of neonatal and adult cardiomyocytes; over-expression of Tip60α and Tip60β in NIH3T3 fibroblasts; methods of morphometric analysis.
(DOC)

Figure S1 **Specificity of the Anti-Tip60 Antibody.** Western blots were used to determine specificity of the anti-Tip60 antibody. Panel **A**, western blot of total heart proteins electrophoretically separated in two-dimensions (iso-electric focusing then SDS/PAGE). The first-dimension (IEF) gel was loaded with 50 µg protein from three month-old adult mouse hearts. Panel **B**, western blot of proteins induced by IPTG in a bacterial *in vitro* translation system containing plasmids encoding GST-Tip60β and GST-MOF fusion proteins. ui  =  uninduced; i  =  induced. Arrows in the Coomassie-stained gel denote positions of GST-Tip60β (left) and GST-MOF (right) proteins. Panel **C**, lysates of HeLa cells transfected with plasmids encoding FLAG-tagged Tip60α and Tip60β isoproteins were separated in duplicate 7.5% acrylamide/SDS gels and blots were reacted with anti-Tip60 (left) or anti-FLAG (right) antibodies.
(TIF)

Figure S2 **The Non-Cardiomyocyte:Cardiomyocyte Ratio Increases during Myocardial Development.** The Tip60 Isoprotein Transition Occurs in non-Cardiomyocytes and in Cardiomyocytes. Panels A and B are Nkx2.5-immunostained (brown) sections from (A) embryonic and (B) adult mouse hearts. Nuclei were counter-stained with hematoxylin (blue). Scale bars  =  20 µm. Panel C shows percentages of Nkx2.5-positive cardiomyocytes. At least 10,000 embryonic and 3,000 adult nuclei were enumerated. Error bars  =  range of mean values from two hearts. Panel D shows cardiomyocytes isolated from eight week-old adult hearts (scale bar  = 30 µm) that were used to prepare the western blot shown in panel E. Panel E is a western blot displaying Tip60α and Tip60β isoprotein levels in cardiomyocytes (CM) and non-cardiomyocytes (non-CM) isolated from two day-old neonatal and eight week-old adult hearts. This blot is representative of four independent cell separations, in each of which bands were densitometrically quantitated and averaged (±SEM) as shown in panel F.
(TIF)

Figure S3 **Expression of MycER Transgene and 4-OHT-Induced Hypertrophy in WT and Het Adult Hearts.** Panel **A** shows expression of the MycER transgene in hearts of eight week-old adult wild type (WT) and Tip60-heterozygous (Het) mice, determined by semi-quantitative RT/PCR; the image is an autoradiograph of ^32^P-labeled PCR products. Panel **B** shows results of densitometry to quantitate the MycER bands shown in **A**. Panel **C** shows the effect of seven days' 4-OHT treatment on heart mass expressed relative to tibia length. n-Tg  =  non-transgenic; error bars  =  ±SEM.
(TIF)

Figure S4 **Trend Toward Reduced Tip60 Protein Levels in Aortic Banded and c-Myc Stressed Myocardium.** Heart protein lysates were isolated and separated on 7.5% acrylamide/SDS gels. Panels **A** and **B** are western blots sequentially reacted with anti-Tip60 and anti-GAPDH antibodies. Panels **C** and **D** respectively show results from quantitative densitometry of bands in **A** and **B**; Tip60 protein levels are normalized to GAPDH. Genotypes in **D** are: ns ** = ** Het/MycER/-4-OHT; WT  =  WT/MycER/+4-OHT; Het  =  Het/MycER/+4-OHT. p-values were calculated by Student's t-test. ns  =  not stressed; nb  =  not banded.
(TIF)

Figure S5 **Echocardiography of 4-OHT-induced Transgenic WT and Tip60-Heterozygous Mice.** Left ventricular (LV) wall thickness and internal diameter were assessed during diastole in isoflurane-anesthestized three month-old mice, at baseline and after eight days' treatment with 4-OHT. Echocardiography using the parasternal long axis view was performed with a VisualSonics Vevo 770 high-frequency ultrasound rodent imaging system. LV mass was calculated at diastole using the following formula: 1.053*((LV internal diameter+posterior wall thickness+inraventricular septum thickness)^3^-LV internal diameter^3^). There were no differences among the experimental groups in measures of cardiac systolic function, including fractional shortening. Each bar indicates the mean value of 6–7 mice. Tip60^+/+^  =  WT; Tip60^+/−^  =  Het MycER.
(TIF)

## References

[1] Sapountzi V, Logan IR, Robson CN (2006). Cellular functions of TIP60.. Int J Biochem Cell Biol.

[2] Pena AN, Tominaga K, Pereira-Smith OM (2011). MRG15 activates the cdc2 promoter via histone acetylation in human cells.. Exp Cell Res.

[3] Yamamoto T, Horikoshi M (1997). Novel substrate specificity of the histone acetyltransferase activity of HIV-1-Tat interactive protein Tip60.. J Biol Chem.

[4] Voss AK, Thomas T (2009). MYST family histone acetyltransferases take center stage in stem cells and development.. Bioessays.

[5] Kim MS, Merlo X, Wilson C, Lough J (2006). Co-activation of atrial natriuretic factor promoter by Tip60 and serum response factor.. J Biol Chem.

[6] Lehner B, Crombie C, Tischler J, Fortunato A, Fraser AG (2006). Systematic mapping of genetic interactions in Caenorhabditis elegans identifies common modifiers of diverse signaling pathways.. Nat Genet.

[7] Berns K, Hijmans EM, Mullenders J, Brummelkamp TR, Velds A (2004). A large-scale RNAi screen in human cells identifies new components of the p53 pathway.. Nature.

[8] Hu Y, Fisher JB, Koprowski S, McAllister D, Kim MS (2009). Homozygous disruption of the Tip60 gene causes early embryonic lethality.. Dev Dyn.

[9] Legube G, Linares LK, Tyteca S, Caron C, Scheffner M (2004). Role of the histone acetyl transferase Tip60 in the p53 pathway.. J Biol Chem.

[10] Tang Y, Luo J, Zhang W, Gu W (2006). Tip60-dependent acetylation of p53 modulates the decision between cell-cycle arrest and apoptosis.. Mol Cell.

[11] Tang Y, Zhao W, Chen Y, Zhao Y, Gu W (2008). Acetylation is indispensable for p53 activation.. Cell.

[12] Gevry N, Chan HM, Laflamme L, Livingston DM, Gaudreau L (2007). p21 transcription is regulated by differential localization of histone H2A.Z.. Genes Dev.

[13] Muckova K, Duffield JS, Held KD, Bonventre JV, Sheridan AM (2006). cPLA2-interacting protein, PLIP, causes apoptosis and decreases G1 phase in mesangial cells.. Am J Physiol Renal Physiol.

[14] Gorrini C, Squatrito M, Luise C, Syed N, Perna D (2007). Tip60 is a haplo-insufficient tumour suppressor required for an oncogene-induced DNA damage response.. Nature.

[15] Ikura T, Ogryzko VV, Grigoriev M, Groisman R, Wang J (2000). Involvement of the TIP60 histone acetylase complex in DNA repair and apoptosis.. Cell.

[16] Sykes SM, Mellert HS, Holbert MA, Li K, Marmorstein R (2006). Acetylation of the p53 DNA-binding domain regulates apoptosis induction.. Mol Cell.

[17] Tyteca S, Legube G, Trouche D (2006). To die or not to die: a HAT trick.. Mol Cell.

[18] Soonpaa MH, Field LJ (1998). Survey of studies examining mammalian cardiomyocyte DNA synthesis.. Circ Res.

[19] Engel FB, Schebesta M, Duong MT, Lu G, Ren S (2005). p38 MAP kinase inhibition enables proliferation of adult mammalian cardiomyocytes.. Genes Dev.

[20] Kuhn B, del Monte F, Hajjar RJ, Chang YS, Lebeche D (2007). Periostin induces proliferation of differentiated cardiomyocytes and promotes cardiac repair.. Nat Med.

[21] Bersell K, Arab S, Haring B, Kuhn B (2009). Neuregulin1/ErbB4 signaling induces cardiomyocyte proliferation and repair of heart injury.. Cell.

[22] Bergmann O, Bhardwaj RD, Bernard S, Zdunek S, Barnabe-Heider F (2009). Evidence for cardiomyocyte renewal in humans.. Science.

[23] Kajstura J, Gurusamy N, Ogorek B, Goichberg P, Clavo-Rondon C (2010). Myocyte turnover in the aging human heart.. Circ Res.

[24] Oh H, Taffet GE, Youker KA, Entman ML, Overbeek PA (2001). Telomerase reverse transcriptase promotes cardiac muscle cell proliferation, hypertrophy, and survival.. Proc Natl Acad Sci U S A.

[25] Lough JW (2002). Transient expression of TIP60 protein during early chick heart development.. Dev Dyn.

[26] Xiao G, Mao S, Baumgarten G, Serrano J, Jordan MC (2001). Inducible activation of c-Myc in adult myocardium in vivo provokes cardiac myocyte hypertrophy and reactivation of DNA synthesis.. Circ Res.

[27] Littlewood TD, Hancock DC, Danielian PS, Parker MG, Evan GI (1995). A modified oestrogen receptor ligand-binding domain as an improved switch for the regulation of heterologous proteins.. Nucleic Acids Res.

[28] McAllister D, Merlo X, Lough J (2002). Characterization and expression of the mouse tat interactive protein 60 kD (TIP60) gene.. Gene.

[29] Gehrking KM, Andresen JM, Duvick L, Lough J, Zoghbi HY (2011). Partial Loss of Tip60 Slows Midstage Neurodegeneration in a Spinocerebellar Ataxia Type 1 (SCA1) Mouse Model.. Hum Mol Genet.

[30] Banerjee I, Fuseler JW, Price RL, Borg TK, Baudino TA (2007). Determination of cell types and numbers during cardiac development in the neonatal and adult rat and mouse.. Am J Physiol Heart Circ Physiol.

[31] Zhang Y, Li TS, Lee ST, Wawrowsky KA, Cheng K (2010). Dedifferentiation and proliferation of mammalian cardiomyocytes.. PLoS One.

[32] Lee Y, Gustafsson AB (2009). Role of apoptosis in cardiovascular disease.. Apoptosis.

[33] Lee HJ, Chun M, Kandror KV (2001). Tip60 and HDAC7 interact with the endothelin receptor a and may be involved in downstream signaling.. J Biol Chem.

[34] Hass MR, Yankner BA (2005). A {gamma}-secretase-independent mechanism of signal transduction by the amyloid precursor protein.. J Biol Chem.

[35] Porrello ER, Mahmoud AI, Simpson E, Hill JA, Richardson JA (2011). Transient regenerative potential of the neonatal mouse heart.. Science.

[36] Izumo S, Nadal-Ginard B, Mahdavi V (1988). Protooncogene induction and reprogramming of cardiac gene expression produced by pressure overload.. Proc Natl Acad Sci U S A.

[37] Legube G, Linares LK, Lemercier C, Scheffner M, Khochbin S (2002). Tip60 is targeted to proteasome-mediated degradation by Mdm2 and accumulates after UV irradiation.. EMBO J.

[38] Ahuja P, Zhao P, Angelis E, Ruan H, Korge P (2010). Myc controls transcriptional regulation of cardiac metabolism and mitochondrial biogenesis in response to pathological stress in mice.. J Clin Invest.

[39] Zhong W, Mao S, Tobis S, Angelis E, Jordan MC (2006). Hypertrophic growth in cardiac myocytes is mediated by Myc through a Cyclin D2-dependent pathway.. EMBO J.

[40] Meckert PC, Rivello HG, Vigliano C, Gonzalez P, Favaloro R (2005). Endomitosis and polyploidization of myocardial cells in the periphery of human acute myocardial infarction.. Cardiovasc Res.

[41] MacLellan WR, Garcia A, Oh H, Frenkel P, Jordan MC (2005). Overlapping roles of pocket proteins in the myocardium are unmasked by germ line deletion of p130 plus heart-specific deletion of Rb.. Mol Cell Biol.

[42] Sdek P, Zhao P, Wang Y, Huang CJ, Ko CY (2011). Rb and p130 control cell cycle gene silencing to maintain the postmitotic phenotype in cardiac myocytes.. J Cell Biol.

[43] Kim KK, Soonpaa MH, Daud AI, Koh GY, Kim JS (1994). Tumor suppressor gene expression during normal and pathologic myocardial growth.. J Biol Chem.

[44] Zhu W, Hassink RJ, Rubart M, Field LJ (2009). Cell-cycle-based strategies to drive myocardial repair.. Pediatr Cardiol.

[45] Sun Y, Jiang X, Xu Y, Ayrapetov MK, Moreau LA (2009). Histone H3 methylation links DNA damage detection to activation of the tumour suppressor Tip60.. Nat Cell Biol.

[46] Narita M, Nunez S, Heard E, Lin AW, Hearn SA (2003). Rb-mediated heterochromatin formation and silencing of E2F target genes during cellular senescence.. Cell.

[47] Pajcini KV, Corbel SY, Sage J, Pomerantz JH, Blau HM (2010). Transient inactivation of Rb and ARF yields regenerative cells from postmitotic mammalian muscle.. Cell Stem Cell.

[48] Sun P, Yoshizuka N, New L, Moser BA, Li Y (2007). PRAK is essential for ras-induced senescence and tumor suppression.. Cell.

