# Supplementary material for: Stress-Induced Cell-Cycle Activation in Tip60 Haploinsufficient Adult Cardiomyocytes
Source: PLoS One. 2012 Feb 14;7(2):e31569. doi: 10.1371/journal.pone.0031569 (PMC3279378; doi:10.1371/journal.pone.0031569)
Supplement: File S1 — This file describes details of the Materials and Methods used for this study. These include animal identity, care and handling; plasmid descriptions; Tip60 antibody characterization; western blotting; antibodies employed & immunohistochemical protocols; semi-quantitative RT-PCR protocols; protocols for induction of cardiac hypertrophy by aortic banding; methods for isolation & culture of neonatal and adult cardiomyocytes; over-expression of Tip60α and Tip60β in NIH3T3 fibroblasts; methods of morphometric analysis. (DOC) [file pone.0031569.s001.doc]

**Supporting Information (File S1)**

***Animals:*** All aspects of this investigation adhered to the NIH Guide for the Care and Use of Laboratory Animals (NIH Pub. No. 85-23, Revised 1996). All protocols, which are described in the corresponding author's Animal Use Application (AUA) #00000225, were approved by the Medical College of Wisconsin Institutional Animal Care and Use Committee (IACUC). The Medical College of Wisconsin has an Animal Welfare Assurance on file with the Office of Laboratory Animal Welfare (A3102-01).

***Plasmids:*** For the transient transfection experiments shown in Figure S1C and in Figure 3, N-terminal FLAG-Tip fusion proteins were prepared by cloning full-length cDNAs encoding *Tip60β* and *Tip60α* into the EcoRI and BamHI sites of expression vector *p3xFLAG-CMV-7.1* (Sigma #E4026). *Tip60β* *cDNA* was from I.M.A.G.E. Consortium clone #3154297 (GenBank accession #AW762337). *Tip60*α mRNA was harvested from mouse testis. For the *in vitro* translations shown in Figure 1B, *GST-Tip60β* plasmid was obtained via generous gift from Dr. Alice M. Sheridan [1], and, GST-MOF was prepared by cloning mouse MOF cDNA I.M.A.G.E. Consortium clone #5136304 (GenBank accession #67773) into pGEX-5X1 vector (GE Healthcare #28-9545-53).

***Tip60 Antibody Characterization:*** Tip60 was detected using an affinity-pure rabbit polyclonal antibody (Bethyl Inc.) that was raised using peptide EGCRLPVLRRNQDNE as antigen; this peptide is present in the N-terminus of all known Tip60 isoproteins, and has no sequence homology with the highly related MYST protein MOF. Specificity of this antibody for Tip60 was confirmed as shown in Figure S1. First, panel A shows that two-dimensional separation (first dimension, iso-electric focusing; second dimension, SDS/PAGE) of total adult heart proteins and immunoblotting with anti-Tip60 detects a single ~53 kD band comprised of differentially charged species at or just below the iso-electric point of Tip60β (pI=8.6); this pattern is consistent with the exclusive presence of un-modified, as well as acetylated and/or phosphorylated forms, of Tip60β in the adult heart. Second (panel B), plasmids containing cDNAs encoding N-terminal fusions of GST with Tip60β and the closely related MYST protein MOF were induced using 0.2 mM isothiopropyl-B-D-galactoside (IPTG) in a bacterial *in vitro* translation system as described in [1], followed by immunoblotting with anti-Tip60. This revealed the presence of a protein migrating at a MW consistent with Tip60β in the reaction containing the GST-Tip60β plasmid (the lower MW fragments are presumably degradation products), but not in the reaction containing GST-MOF plasmid. Third (panel C), plasmids encoding N-terminal FLAG tags of Tip60α and Tip60β cDNAs (described in [2]) were transfected into HeLa cells, followed by immunoblotting parallel samples with anti-Tip60 and anti-FLAG (Sigma #F3165) antibodies. Detection of identically sized bands with both antibodies indicates that anti-Tip60 recognized its cognate protein.

***Western Blotting:*** Mice were anesthetized with 4% isoflurane, the chest wall was removed and the heart was rapidly perfused via gravity (100 mm) using 20 ml ice-cold cardioplegic solution (25 mM KCl/5% dextrose/1x PBS) introduced via the apex of the left ventricle with a 27-gauge needle. Hearts were rapidly sectioned, transversely along the midpoint of the superior-inferior axis. The part of the superior half of the left ventricle was minced with scissors, sonicated, and suspended in lysis buffer (20mM HEPES [pH 7.5]/150mM NaCl/1mM EDTA/100mM dithiothreitol/1% Triton-X-100/5% glycerol) including 1x protease inhibitor cocktail (Roche #1836153). Solubilized samples were assessed for protein concentration using Bradford assay (Bio-Rad #500-0006) and stored at -80°C until electrophoresis. For one-dimensional electrophoresis, samples containing exactly 10 μg total protein were suspended in SDS sample buffer (250mM Tris-HCl [pH 6.8]/3.75% glycerol/0.005% bromphenol blue/2% SDS/100mM dithiothreitol), heated at 100°C for 5 minutes, and electrophoretically separated on 7.5% acrylamide:bis (30:0.8)/SDS gels (Bio-Rad #161-1118). Separated proteins were electroblotted onto a nitrocellulose membrane (Bio-Rad #162-0146) and blocked overnight with NFDM/TBST (5% non-fat dry milk/10mM Tris-HCl (pH 7.6)/150mM NaCl/0.05% Tween-20). Blots were sequentially reacted with the following primary antibodies: anti-Tip60 (1:1,000), anti-cyclin D2 (Santa Cruz Biotech #sc-593; 1:250), and anti-GAPDH (Advanced Immunochemicals #6C5; 1:1,000) diluted in blocking buffer overnight at 4°C. Secondary antibodies were goat anti-rabbit IgG horseradish peroxidase conjugate (Bio-Rad #170-6515; 1:7,500) for anti-Tip60 and anti-cyclin D2, and goat anti-mouse IgG horseradish peroxidase conjugate (Bio-Rad #170-6516; 1:7,500) for anti-GAPDH. Secondary antibodies were diluted in NFDM/TBST and applied for 60 minutes at RT. Reacted blots were covered with HRP-substrate (Amersham #RPN2106) for 1 minute at room temperature followed by antigen localization performed by exposing the membrane to Hyperfilm-ECL (Kodak #8294985) for intervals up to 5 minutes. Film was processed in a Kodak X-Omat 2000A developer. Films were scanned using a Hewlitt-Packard Scanjet G4010 scanner. Densitometry was performed using ImageJ software.

***Antibodies & Immunohistochemistry:*** To immunostain embryonic and adult cardiomyocytes *in vivo*, hearts were fixed by immersion in freshly prepared 4% paraformaldehyde/PBS, overnight at 4^o^C. After thorough washing, specimens were embedded in Paraffin, followed by placement of 4 μm sections on microscope slides. Cultures of neonatal cardiomyocytes were rinsed with PBS and fixed overnight at 4 ^o^C using fresh 4% paraformaldehyde/PBS (Fig. 4 only).

The immunostains shown in Figures 4 and 8 were performed as follows. Sections were de-waxed (Fig. 8 only), subjected to antigen retrieval by heating (99 ^o^C) in citrate buffer (pH 6.1; below) for 20 min followed by 15 min cooling, and blocked for 60 min at RT with 5% non-fat dry milk/2% goat serum/0.2% Triton-X-100/PBS. Sources and dilutions of the primary antibodies were: (i) activated Caspase-3 (BD Pharmingen rabbit IgG #559565; 1:100); (ii) Ki-67 (Santa Cruz goat IgG #sc-7846; 1:500); (iii) sarcomeric α-actin (Sigma mouse IgM #A2172; 1:800). Primary antibodies were applied in blocking buffer overnight at 4°C. Respective secondary antibodies were: (i) Vector Laboratories biotinylated goat anti-rabbit IgG #BA-1000 (15 μl/ml); (ii) Vector Laboratories biotinylated rabbit anti-goat IgG #BA-5000 (15 μl/ml); (iii) ICN fluorescent goat anti-mouse IgM #55498 (1:200). Secondary antibodies were applied in blocking buffer for one hour at RT. After thorough washing, signal for Caspase-3 or Ki-67 was visualized by sequential treatment with Elite ABC-Peroxidase Reagent (Vector Laboratories #PK-7100) and DAB Substrate Kit (Thermo Scientific, Rockford IL #34002). Nuclei were counter-stained with DAPI (Sigma, #D9542; 1 ng/ml). Light microscopic and epifluorescent evaluation of staining was made using a Nikon Eclipse TE300 microscope.

The immunostains shown in Figure S2, and in Figures 5 and 6, were performed as follows. Sections were de-waxed, subjected to antigen retrieval by heating (99 ^o^C) in citrate buffer (pH 6.1; Target Retrieval Solution; DAKO #S1699) for 20 min followed by 15 min cooling, and sequentially treated with PeroxAbolish (Biocare Medical, Concord CA, #PXA969; 15 min) and Avidin/Biotin Blocking Kit (Vector Laboratories #SP-2001; 10 min each). Immunostaining Nkx2.5 was preceded by blocking with Protein Block Serum-Free (DAKO #X0909); the primary antibody was R&D Systems goat IgG (#AF2444; 1:75), which was followed by sequential application of biotinylated mouse anti-goat IgG (Biocare #MG610), streptavidin-HRP (Biocare #HP604), and DAB+ (DAKO #K3468). Immunostaining phosphorylated histone H3 (H3P) was preceded by blocking with Protein Block Serum-Free (DAKO #X0909); the primary antibody was Millipore rabbit IgG (#06-570; 1:500), followed sequentially by 4plus biotinylated goat anti-rabbit IgG (Biocare #GR608), streptavidin-HRP (Biocare #HP604), and DAB+ (DAKO #K3468). For fluorescent detection of H3P (Fig. 7B,E) the secondary antibody was goat anti-rabbit 488 AlexaFluor #A-11008 (1:500). Immunostaining BrdUrd was preceded by blocking endogenous mouse IgG with Rodent Block M (Biocare #RBM961; 30 min); the primary antibody was Developmental Studies Hybridoma Bank mouse IgG_1_ (DSHB #G3G4; 1:100), followed by treatment with Mouse-on-Mouse HRP-Polymer (Biocare #HM620) and DAB+ (DAKO #K3468). Aurora kinase B (AIM-1) was localized identical to BrdUrd except that the primary antibody was BD Biosciences mouse IgG_1_ (#611082; 1:50). Following all immunostains, nuclei were counter-stained with Mayer’s hematoxylin (DAKO #S3309) for 1 min at RT followed by bluing. Light microscopic and epifluorescent observations were made using a Nikon Eclipse TE300 microscope.

In all instances, at least 1,500 cells in both ventricles were enumerated to determine percentages of labeled cells, calculated in reference to the total number of nuclei counter-stained with hematoxylin or DAPI. Statistical significance was determined by subjecting data to Student’s t-Test (two-tailed, unpaired).

***Semi-Quantitative RT-PCR:*** All reactions were performed per manufacturers’ recommendations. Total RNA was purified from heart tissue using RNeasy Plus Mini Kit (Qiagen #74134) followed by quantitation at A_260_. First-strand cDNA was reverse-transcribed from 1.0 μg total RNA using iScript cDNA Synthesis Kit (Bio-Rad #170-8890). PCR was performed using 1/20 of the reverse transcription product as template, in buffer that included 1.5mM MgCl_2_/200μM each nucleotide/0.5μM each primer; for radioactive PCR ~1 µCi α-^32^P-dATP was included. Sequences of the Tip60 primer pair used for the RT-PCR determination shown in Fig. 1A (upper) were 5’-CAGGACAGCTCAGATGGAATACCGTCAGCACCA-3’ [fwd] and 5’-AGAGGACGGGTAGCGTGGTAAG-3’ [rev], which amplifies a 184bp PCR product from either Tip60α or Tip60β. Sequences of the Tip60 primer pair used for the RT-PCR determination shown in Fig. 1A (lower) were 5’-GAGATAATCGAGGGCTGCCG-3’ [fwd] and 5’-ATTAGATTTCCGCTTCCGTCCAGG-3’ [rev], which anneal to exons that flank exon 5 (which is deleted during processing of Tip60β mRNA) respectively amplify 558 and 402 bp bands from Tip60α and Tip60β. Sequences of the Tip60 primer pair used for the RT-PCR determination shown in Figure 2 were 5’-GCCTGGACGGAAGCGGAAATCTAAT-3’ [fwd] and 5’-AAACACTTGGCCAGAAGACACAG-3’ [rev], amplifying a 420bp PCR product from either Tip60α or Tip60β. In Fig. 1A, mouse GAPDH cDNA was amplified using 5’-TGGCAAAGTGGAGATTGTTGCC-3’ (fwd) and 5’-AAGATGGTGATGGGCTTCCCG-3’ (rev), yielding a 156bp PCR product. In Figure 2, GAPDH cDNA was amplified using 5’-ATGGTGAAGGTCGGTGTGAA-3’ (fwd) and 5’-TGGTGGTGCAGGATGCATTG-3’ (rev), yielding a 450bp PCR product. In Fig. S3, MycER transgene cDNA was amplified using 5’-AACGCCTTTGCTGCTCTTGTCA-3’ (fwd) and 5’-GACGATCCAACCAGTTATTCGG-3’ (rev), yielding a 277bp PCR product. PCR was initiated with Go*Taq* DNA Polymerase (Promega #M1661). Non-radioactive PCR products were separated on a 1% agarose gel followed by staining with ethidium bromide. Radioactive PCR reactions were terminated at increasing cycle numbers to ensure that products were in linear amplification range, followed by separation on 5% acrylamide/TBE gels which were dehydrated, exposed to a phosphorimager screen for 4 hours, and developed using a Storm 820 scanner (GE Healthcare #92396). Bands were densitometrically analyzed with ImageJ software.

***Induction of Cardiac Hypertrophy by Aortic Banding:*** To perform aortic banding, surgical procedures were performed under standard aseptic conditions. Adult mice 12-14 weeks old were injected with intraperitoneal barbital (100 mg/kg) to induce anesthesia. After endotracheal intubation with a PE-60 polyethylene tube connected to a mouse ventilator (Hugo Sachs Elektronic Model 845, Germany), respirations were maintained (tidal volume = 225 μL; rate – 100 strokes/min) using room air supplemented with 100% oxygen. A small incision was made at the level of the suprasternal notch and the sternum was opened ~3 mm to expose the thymus. Using gentle micro-dissection, the transverse aorta was exposed and aligned next to a 27 gauge needle. The aorta and needle were ligated together by passing a 7-0 prolene suture between the innominate and left common carotid arteries; after ligation, the needle was gently removed, leaving a constricted aorta with a luminal diameter of ~0.4 mm. The chest was closed with polypropylene sutures and the mice were removed from the ventilator. The mice were placed in a warmed chamber flushed with oxygen until they fully recovered from the surgery. Within two weeks, this procedure resulted in a 45% increase in left ventricular mass.

***Isolation & Culture of Neonatal and Adult Cardiomyocytes:*** Neonatal cardiomyocytes were harvested using the Worthington (#LK003300) Neonatal Cardiomyocyte Isolation System. Briefly, 2-3 day old neonates were euthanized, their hearts were removed and minced, followed by enzymatic digestion in trypsin (0.5 µg/µl) overnight at 4 ^o^C. After terminating tryptic digestion with soybean trypsin inhibitor, digestion was continued with collagenase type II (300 U/ml) for 45 minutes at 37°C. Digested tissue was triturated and large chunks were allowed to settle at 1 x g. The supernatant was discarded and L15 medium was added, followed by trituration, 5 minutes’ continued digestion and centrifugation at 100 x g. The pelleted cells were re-suspended in 117mM NaCl/1mM NaH_2_PO_4_/5.6mM glucose/5.4mM KCl/0.8 mMMgSO_4_/20mM HEPES (pH 7.4) buffer (Ads), layered on a Percoll (Sigma P4937) step gradient (top = 40.5%; bottom=58.5%) prepared in Ads buffer, and centrifuged (1,900 x g 30 minutes) at RT. Non-cardiomyocytes sedimenting at the interface above the top (40.5%) Percoll were saved as the non-cardiomyocyte fraction (Fig. S2). Cardiomyocytes sedimenting at the interface above the bottom (58.5%) Percoll were further enriched by removing remaining fibroblasts, which preferentially attach to uncoated tissue culture dishes (Corning TC-treated polystyrene) during a 1-hour incubation at 37°C. Cardiomyocytes in the supernatant were either washed with PBS and processed for western blotting as described in Figure S2, or cultured as described in Figure 4. Neonatal cardiomyocytes were grown on 1.0% gelatin-coated dishes in standard neonatal cardiomyocyte medium (335 ml DMEM/85 ml Medium 199/50 ml horse serum/25 ml calf serum/5 mL L-glutamine/5 mL penicillin/streptomycin), with daily medium changes.

Adult cardiomyocytes were isolated by cannulation/perfusion as previously described (AFCS Procedure Protocol PP00000125). All buffers used for this procedure were made from stock perfusion buffer consisting of: 113mM NaCl/4.7mM KCl/0.6mM KH_2_HPO_4_/0.6mM Na_2_HPO_4_/1.2mM MgSO_4_-7H_2_O/12mM NaHCO_3_/10mM KHCO_3_/10mM HEPES/30mM taurine/0.032mM phenol red/10mM 2, 3-butanedione monoxime/5.5mM glucose. Approximately 8 week-old mice were euthanized by IP pentobarbital injection and the thoracic cavity was opened. Hearts were removed, a cannula was inserted into the aorta, and perfusion was performed by introducing myocyte digestion buffer pre-warmed at 37°C – 0.14mg/ml trypsin (Invitrogen #15090046)/0.25mg/ml liberase blendzyme (Roche #1988417)/12.5μM CaCl_2_ – which was perfused at a rate of 3 ml/min for 8 minutes. This was followed by sequential perfusion using pre-warmed (37°C) myocyte stopping buffer #1 (stock perfusion buffer/10% bovine calf serum/12.5μM CaCl_2_), and myocyte stopping buffer #2 (stock perfusion buffer/5% bovine calf serum/12.5μM CaCl_2_). Hearts were removed from the cannula and minced with tweezers and tissue forceps. The tissue was disrupted by gentle pipetting and liberated cells were pelleted by centrifugation. The pelleted cells were resuspended in myocyte plating medium (MEM [Gibco #10370]/5% bovine calf serum/10 mM BDM/100 U/ml penicillin/2 mM L-glutamine) and pre-plated on 0.1% gelatin-coated dishes for 6 hours. The unattached rod-shaped myocytes, and adherent non-myocytes, were washed with PBS, flash-frozen in liquid nitrogen and processed for western blotting.

***Over-Expression of Tip60α and Tip60β in NIH3T3 Fibroblasts:*** Tip60α and Tip60β cDNAs were sub-cloned into p3X-CMV-FLAG vector (Sigma #E7533) and recombinant plasmids and empty vector were purified using an Endotoxin Free Maxi Kit (Qiagen #12362) per the manufacturer’s protocol. NIH/3T3 cells (ATCC #CRL-1658) were maintained according to the American Type Culture Collection recommended protocol. For plasmid transfection, two volumes of trypsinized cells re-suspended in antibiotic-free NIH/3T3 medium at a concentration of 375,000 cells/ml were mixed with one volume of Lipofectamine 2000 (Invitrogen #11668) transfection medium containing the plasmid DNA prepared according to the manufacturer’s protocol. Aliquots of 0.6 ml of the cell/plasmid mixture, each of which contained 1.5 μg plasmid DNA, were plated into wells of a 12-well plate that had been pre-coated with 0.1% gelatin. Following overnight (18 hrs) culture at 37 ^o^C, during which time the cells adhered to the culture dish, the transfection medium was exchanged with 0.6 ml antibiotic-free NIH/3T3 medium; an additional medium change was performed three days post-transfection. Five days post-transfection, cells were harvested with Accutase (Innovative Cell Technologies #AT104), followed by enumeration of viable cells using a Guava Easy-cyte Mini cell counter (Millipore #055-1430), and preparation of samples for western blot analysis.

***Morphometric Analysis*** was performed on light microscopic images of cardiomyocytes within the left ventricle at the level of the papillary muscles. Images of immunostained slides were processed using a Hamamatsu Nanozoomer 2.0-HT Slide Scanner, followed by quantitation of two-dimensional area using the freehand area annotation function of this instrument. To estimate cardiomyocyte size, laminin-stained boundaries of individual cells in transverse section through the level of the Nkx2.5-positive myonuclei were outlined, followed by calculation of the circumscribed area. To estimate nuclear density, areas of longitudinally oriented cells were selected and outlined, within which numbers of Nkx2.5-positive nuclei were enumerated; a minimum of 500 nuclei were evaluated in each heart.

**References**

[1] Muckova K, Duffield JS, Held KD, Bonventre JV, Sheridan AM. cPLA2-interacting protein, PLIP, causes apoptosis and decreases G1 phase in mesangial cells. Am J Physiol Renal Physiol. 2006 Jan; 290(1): F70-9.

[2] Kim MS, Merlo X, Wilson C, Lough J. Co-activation of atrial natriuretic factor promoter by Tip60 and serum response factor. J Biol Chem. 2006 Jun 2; 281(22): 15082-9.
