# Supplementary figures and images for: Stress-Induced Cell-Cycle Activation in Tip60 Haploinsufficient Adult Cardiomyocytes
Source: PLoS One. 2012 Feb 14;7(2):e31569. doi: 10.1371/journal.pone.0031569 (PMC3279378; doi:10.1371/journal.pone.0031569)

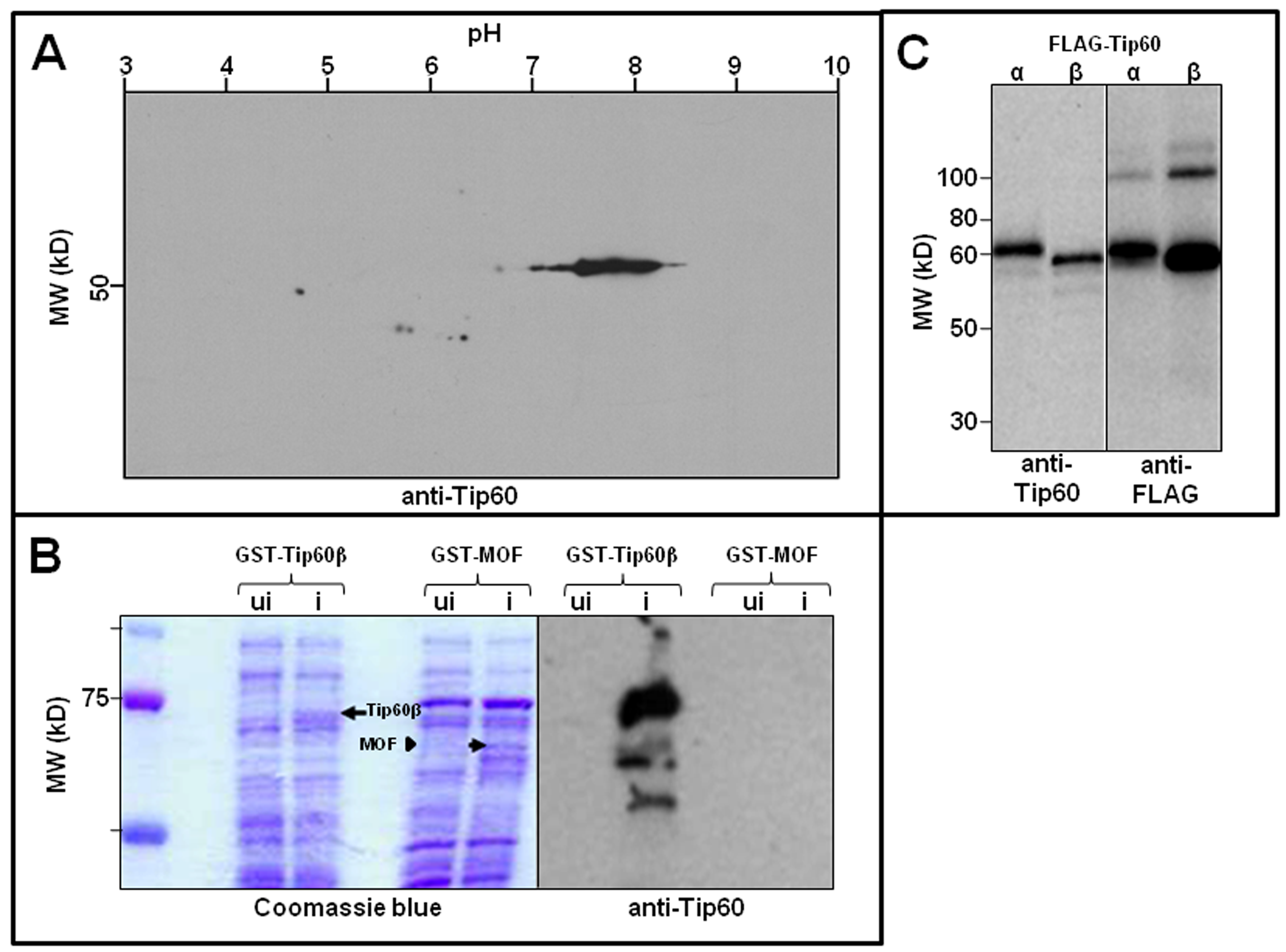

Supplement: Figure S1 — Specificity of the Anti-Tip60 Antibody. Western blots were used to determine specificity of the anti-Tip60 antibody. Panel A, western blot of total heart proteins electrophoretically separated in two-dimensions (iso-electric focusing then SDS/PAGE). The first-dimension (IEF) gel was loaded with 50 µg protein from three month-old adult mouse hearts. Panel B, western blot of proteins induced by IPTG in a bacterial in vitro translation system containing plasmids encoding GST-Tip60β and GST-MOF fusion proteins. ui = uninduced; i = induced. Arrows in the Coomassie-stained gel denote positions of GST-Tip60β (left) and GST-MOF (right) proteins. Panel C, lysates of HeLa cells transfected with plasmids encoding FLAG-tagged Tip60α and Tip60β isoproteins were separated in duplicate 7.5% acrylamide/SDS gels and blots were reacted with anti-Tip60 (left) or anti-FLAG (right) antibodies. (TIF) [file pone.0031569.s002.tif]

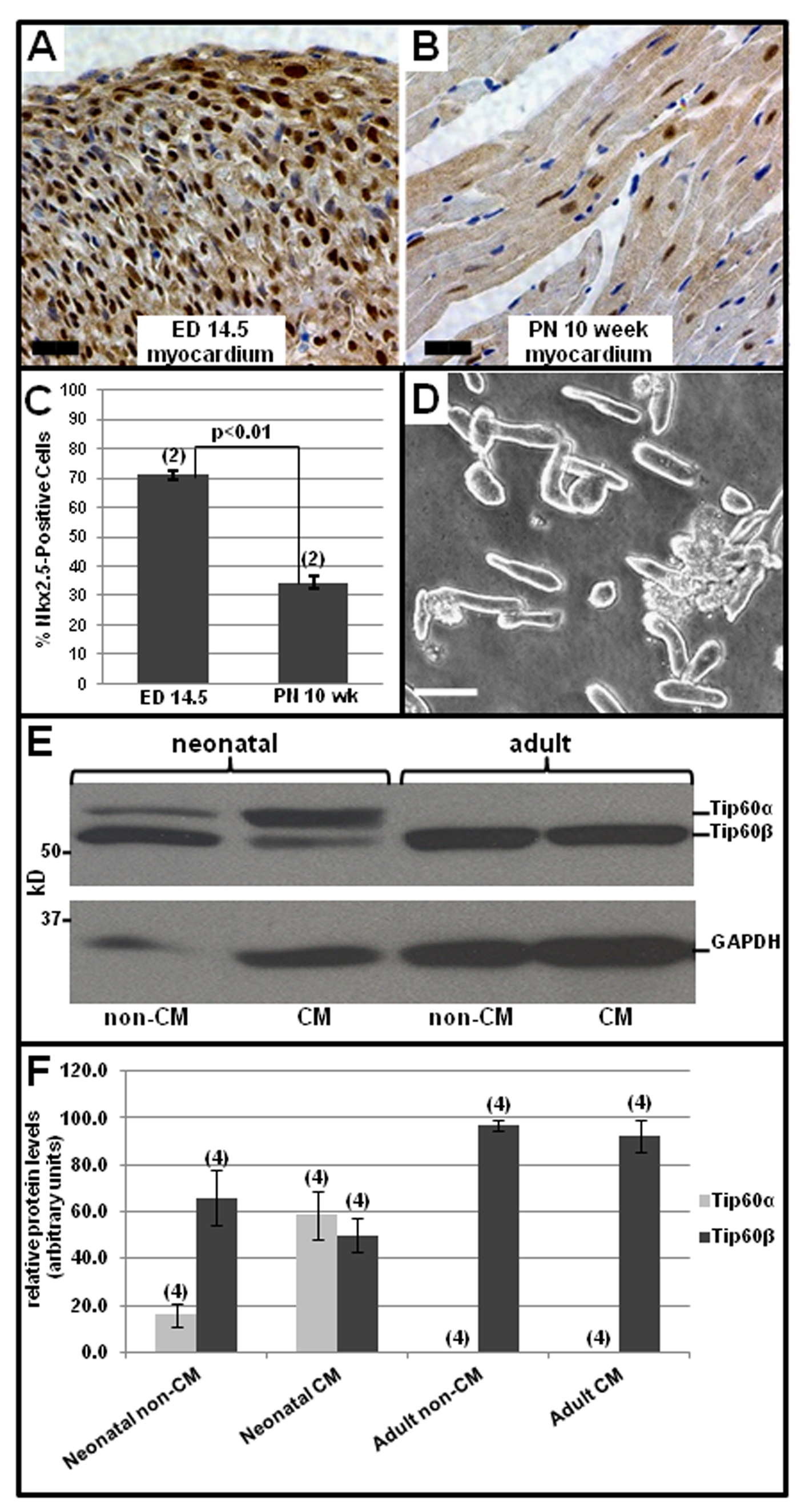

Supplement: Figure S2 — The Non-Cardiomyocyte:Cardiomyocyte Ratio Increases during Myocardial Development. The Tip60 Isoprotein Transition Occurs in non-Cardiomyocytes and in Cardiomyocytes. Panels A and B are Nkx2.5-immunostained (brown) sections from (A) embryonic and (B) adult mouse hearts. Nuclei were counter-stained with hematoxylin (blue). Scale bars = 20 µm. Panel C shows percentages of Nkx2.5-positive cardiomyocytes. At least 10,000 embryonic and 3,000 adult nuclei were enumerated. Error bars = range of mean values from two hearts. Panel D shows cardiomyocytes isolated from eight week-old adult hearts (scale bar = 30 µm) that were used to prepare the western blot shown in panel E. Panel E is a western blot displaying Tip60α and Tip60β isoprotein levels in cardiomyocytes (CM) and non-cardiomyocytes (non-CM) isolated from two day-old neonatal and eight week-old adult hearts. This blot is representative of four independent cell separations, in each of which bands were densitometrically quantitated and averaged (±SEM) as shown in panel F. (TIF) [file pone.0031569.s003.tif]

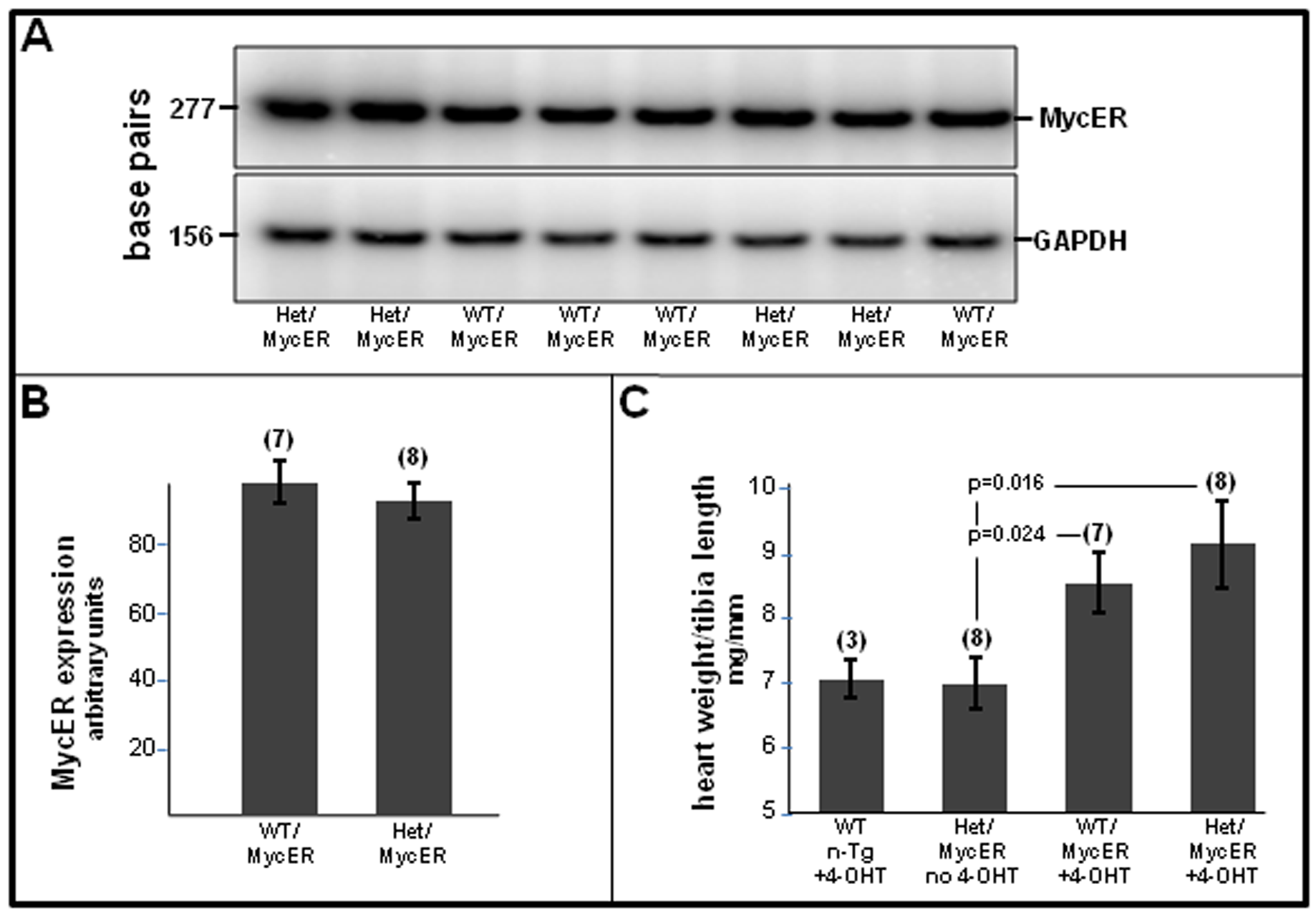

Supplement: Figure S3 — Expression of MycER Transgene and 4-OHT-Induced Hypertrophy in WT and Het Adult Hearts. Panel A shows expression of the MycER transgene in hearts of eight week-old adult wild type (WT) and Tip60-heterozygous (Het) mice, determined by semi-quantitative RT/PCR; the image is an autoradiograph of 32P-labeled PCR products. Panel B shows results of densitometry to quantitate the MycER bands shown in A. Panel C shows the effect of seven days' 4-OHT treatment on heart mass expressed relative to tibia length. n-Tg = non-transgenic; error bars = ±SEM. (TIF) [file pone.0031569.s004.tif]

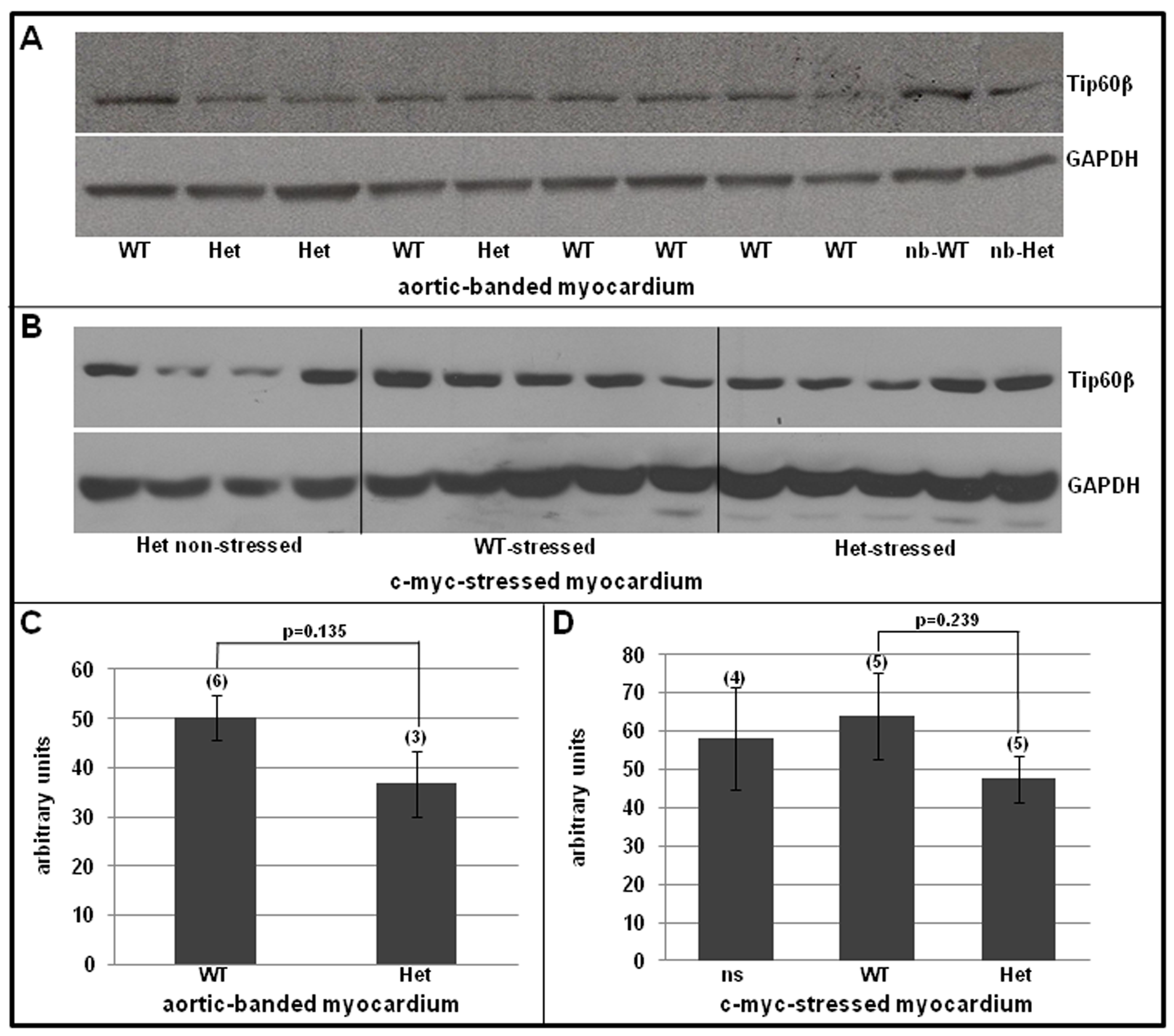

Supplement: Figure S4 — Trend Toward Reduced Tip60 Protein Levels in Aortic Banded and c-Myc Stressed Myocardium. Heart protein lysates were isolated and separated on 7.5% acrylamide/SDS gels. Panels A and B are western blots sequentially reacted with anti-Tip60 and anti-GAPDH antibodies. Panels C and D respectively show results from quantitative densitometry of bands in A and B; Tip60 protein levels are normalized to GAPDH. Genotypes in D are: ns = Het/MycER/-4-OHT; WT = WT/MycER/+4-OHT; Het = Het/MycER/+4-OHT. p-values were calculated by Student's t-test. ns = not stressed; nb = not banded. (TIF) [file pone.0031569.s005.tif]

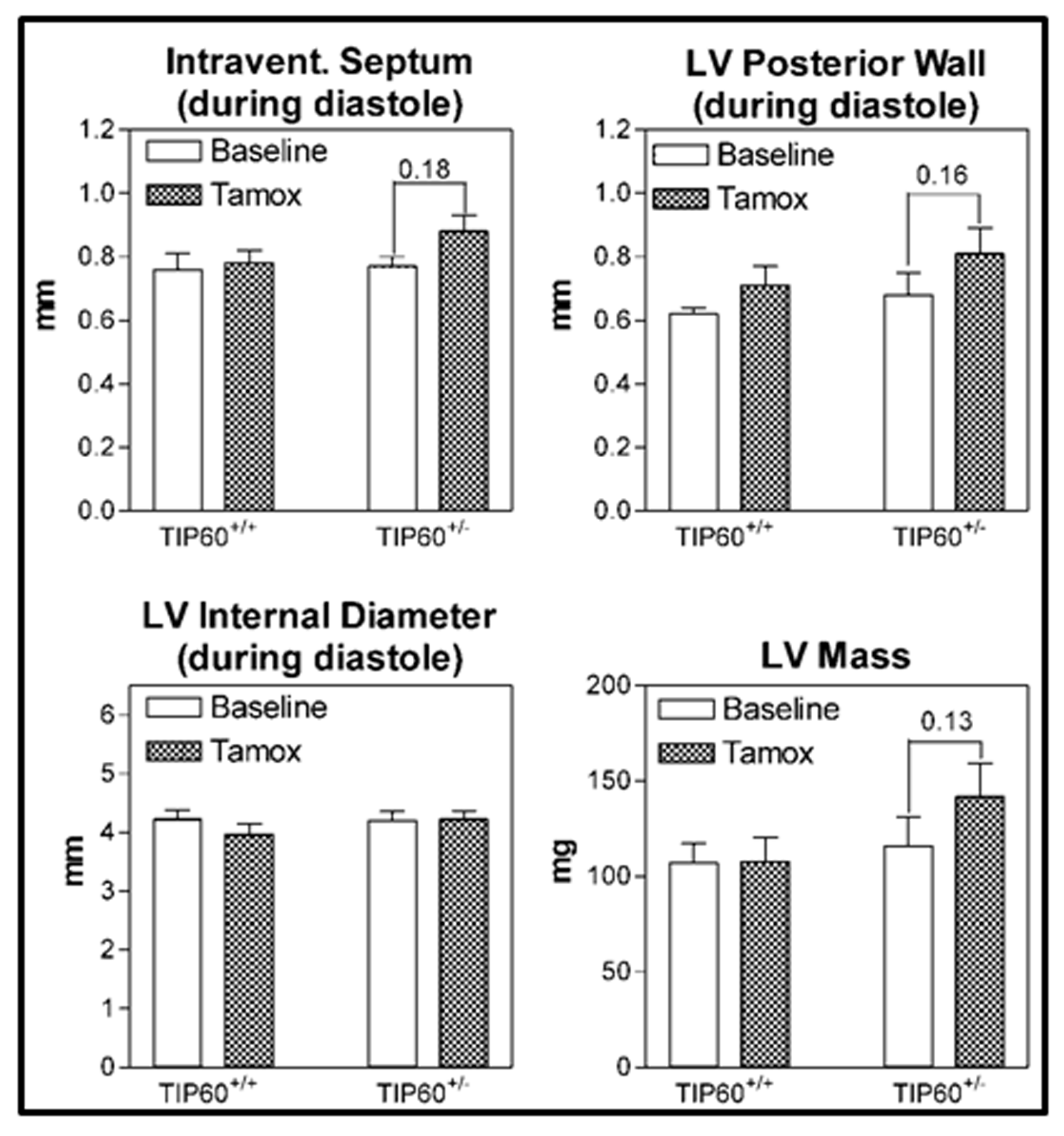

Supplement: Figure S5 — Echocardiography of 4-OHT-induced Transgenic WT and Tip60-Heterozygous Mice. Left ventricular (LV) wall thickness and internal diameter were assessed during diastole in isoflurane-anesthestized three month-old mice, at baseline and after eight days' treatment with 4-OHT. Echocardiography using the parasternal long axis view was performed with a VisualSonics Vevo 770 high-frequency ultrasound rodent imaging system. LV mass was calculated at diastole using the following formula: 1.053*((LV internal diameter+posterior wall thickness+inraventricular septum thickness)3-LV internal diameter3). There were no differences among the experimental groups in measures of cardiac systolic function, including fractional shortening. Each bar indicates the mean value of 6–7 mice. Tip60+/+ = WT; Tip60+/− = Het MycER. (TIF) [file pone.0031569.s006.tif]
